# Targeting of EGFR by a combination of antibodies mediates unconventional EGFR trafficking and degradation

**DOI:** 10.1038/s41598-019-57153-9

**Published:** 2020-01-20

**Authors:** Sylwia Jones, Peter J. King, Costin N. Antonescu, Michael G. Sugiyama, Amandeep Bhamra, Silvia Surinova, Nicos Angelopoulos, Michael Kragh, Mikkel W. Pedersen, John A. Hartley, Clare E. Futter, Daniel Hochhauser

**Affiliations:** 1 Cancer Research UK Drug-DNA Interactions Research Group, UCL Cancer Institute, Paul O’Gorman Building, University College London, London, WC1E 6DD UK; 2 Department of Cell Biology, Ryerson University, Toronto, Canada; 3 Proteomics Research Core Facility, UCL Cancer Institute, University College London, London, UK; 4 Symphogen A/S, Ballerup, Denmark; 5 UCL Institute of Ophthalmology, University College London, 11-43 Bath Street, London, EC1V 9EL UK

**Keywords:** Growth factor signalling, Lysosomes, Ubiquitylation, Membrane trafficking, Endocytosis, Targeted therapies

## Abstract

Antibody combinations targeting cell surface receptors are a new modality of cancer therapy. The trafficking and signalling mechanisms regulated by such therapeutics are not fully understood but could underlie differential tumour responses. We explored EGFR trafficking upon treatment with the antibody combination Sym004 which has shown promise clinically. Sym004 promoted EGFR endocytosis distinctly from EGF: it was asynchronous, not accompanied by canonical signalling events and involved EGFR clustering within detergent-insoluble plasma mebrane-associated tubules. Sym004 induced lysosomal degradation independently of EGFR ubiquitylation but dependent upon Hrs/Tsg101 that are required for the formation of intraluminal vesicles (ILVs) within late endosomes. We propose Sym004 cross-links EGFR physically triggering EGFR endocytosis and incorporation onto ILVs and so Sym004 sensitivity correlates with EGFR numbers available for binding, rather than specific signalling events. Consistently Sym004 efficacy and potentiation of cisplatin responses correlated with EGFR surface expression in head and neck cancer cells. These findings will have implications in understanding the mode of action of this new class of cancer therapeutics.

## Introduction

Dysregulated receptor tyrosine kinase (RTK) signalling is associated with cancer and targeting of such signalling can be effective in cancer therapy. The Epidermal Growth Factor Receptor (EGFR)-targeting monoclonal antibodies (mAbs) cetuximab and panitumumab have been widely used for treatment of *KRAS* wild-type colorectal cancer, whereas the HER2-targeting antibody trastuzumab has been successful in *HER2*-amplified breast cancers^1,2^. Despite clinical benefit of these mAbs, tumours eventually become resistant and relapse.

Multi-epitope targeting by combinations of two or more antibodies against non-overlapping epitopes on the same receptor has been demonstrated to have increased anti-tumour activity compared to individual mAbs in preclinical studies as well as in the clinic^3,4^. Similarly to individual mAbs, the antibody combinations inhibit ligand binding and pro-survival signalling, but in addition promote potent removal of their target receptors^4–8^. However, little is known regarding the mechanisms accounting for the internalization and degradation induced by such antibody combinations.

In the current work we set out to explore in detail the mechanism of RTK internalisation and degradation induced by antibody combinations. The dual epitope targeting anti-EGFR antibody combination Sym004, which has shown therapeutic efficacy in colorectal cancer and is currently being trialled in randomised studies, was used in this work^9–11^. We found that Sym004 promoted EGFR clustering within detergent-insoluble tubules at the cell surface, leading to slow and asynchronous endocytosis in the absence of EGFR tyrosine kinase activity or downstream signalling. The Sym004-EGFR clusters were subsequently targeted for lysosomal degradation independently of EGFR ubiquitylation, but dependent upon the functionality of the ESCRT machinery required for the formation of intraluminal vesicles (ILVs) within late endosomes.

We propose that cross-linking of multiple EGFR molecules within detergent-insoluble plasma membrane clusters physically triggers unconventional EGFR trafficking, *i.e*. prolonged and asynchronous EGFR endocytosis and degradation, without the requirement for the canonical signalling events, and so Sym004-mediated EGFR degradation correlates primarily with the accessibility of EGFR molecules at the plasma membrane/lower proportion of endosomal EGFR unavailable for Sym004 binding. In favour of this model we showed that Sym004 treatment was much more potent in head and neck cancer (HNC) cells with higher expression of cell surfaceEGFR, and Sym004 additionally potentiated response to chemotherapy in these cells.

## Results

### Sym004, but not cetuximab, promotes EGFR internalisation and trafficking

**Figure 1:**
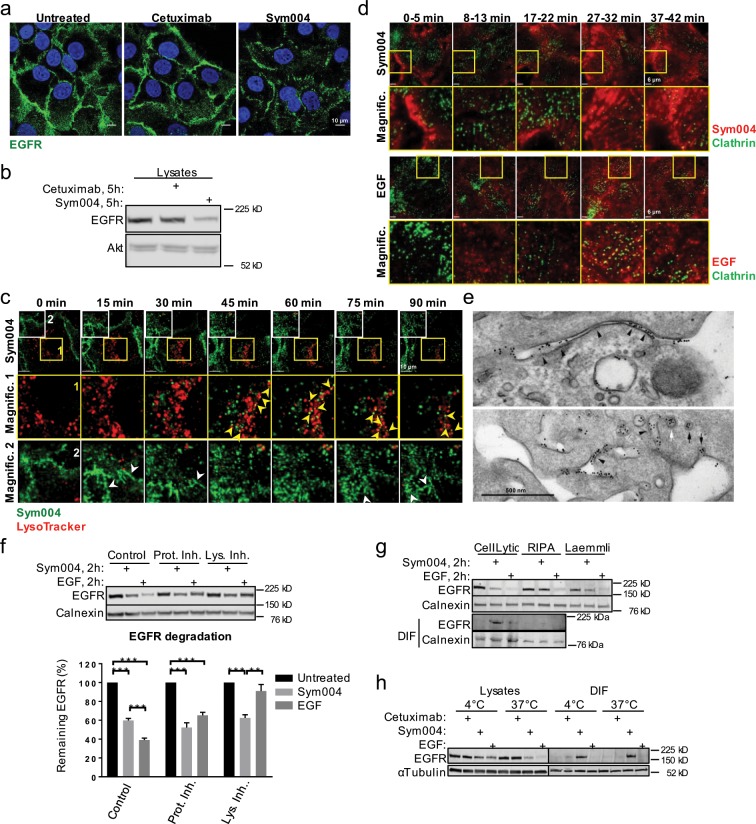
Sym004 forms distinctive cell surface structures and promotes EGFR localisation within a detergent-insoluble fraction. **(a)** SCC47 cells were serum-starved for 2 h, then incubated with cetuximab or Sym004 overnight, fixed and immunostained with α-EGFR antibody. Nuclei stained with Hoechst 33342. **(b)** SCC47 cells were serum-starved for 2 h and treated with cetuximab or Sym004 for 5 h, then lysed with CellLytic M. **(c)** SCC47 cells were serum-starved, final 2 h in the presence of LysoTracker Red (100 nM), then imaged live in cell-imaging medium (CIM) upon addition of Sym004-AF488. The images were acquired every 3 min and generated from 9 z-stacks with 0.424 µm intervals. Yellow arrowheads indicate colocalisation between Sym004 and LysoTracker and white arrowheads point to the elongated tubular structures. **(d)** GFP-CLC cells were serum-starved for a minimum 2 h and imaged live in the presence of EGF-Cy3b. For Sym004, serum-starved cells were pre-treated for 5 min with Sym004-AF568 (10 µg/ml), washed with PBS and imaged for indicated times. **(e)** Cells were treated with Sym004-10 nm Gold for 15 min before processing for electron microscopy. Gold was found in a variety of tubules connected with the cell surface (arrowheads), as well as pits and vesicles (arrows) both coated (white) and uncoated (black). **(f)** SCC47 cells were serum-starved, final 1 h in the presence of MG132 (10 µM), bafilomycin A1 (600 nM) or DMSO (control), then treated with Sym004 or EGF for 2 h. The cells were lysed with CellLytic M. For quantifications, EGFR was normalised to calnexin. **(g)** Serum-starved SCC47 cells were treated with Sym004 or EGF for 2 h, then lysed with different buffers. **(h)** Serum-starved SCC47 cells were incubated on ice with cetuximab, Sym004 or EGF for 30 min. The cells were then either lysed (CellLytic M) or placed for further 2 h at 37 °C, then lysed. Error bars, SEM, **p < 0.01, ***p < 0.001. DIF, detergent-insoluble fraction (remaining after centrifugation of lysates). Nuclei stained with Hoechst 33342. All immunoblots were cropped for clarity.

It has been shown previosuly that cetuximab triggers minimal EGFR endocytosis, which is significantly enhanced in the presence of an additional anti-human antibody^5,7,12^. Consistent with these observations, we did not detect significant EGFR endocytosis following cetuximab treatment in HNC SCC47 cells (Fig. 1a). In contrast, Sym004 triggered noticeable EGFR internalisation and a decrease in EGFR protein levels (Fig. 1a,b). Live-cell imaging further revealed that fluorescently-labelled Sym004 partially colocalised with LysoTracker-positive late endosomes/lysosomes within 90 min of treatment, although the majority of Sym004 remained at, or in the proximity of the plasma membrane, where it formed distinctive elongated structures (Fig. 1c).

To investigate these structures in more detail, we next took advantage of RPE cells stably expressing GFP-clathrin^13^ and analysed the distribution of Sym004 or EGF at the plasma membrane through live-cell total-internal reflection fluorescent microscopy (TIRFM). As expected, EGF displayed punctate localisation and it greatly colocalised with clathrin. In contrast, cell surface structures formed by Sym004 were elongated (Fig. 1d) and there was significantly less colocalisation between Sym004 and clathrin (Supplementary Fig. S1a). We then analysed the subcellular distribution of gold-labelled Sym004 *via* electron microscopy and found that it was present within cell surface-connected tubules at 15 min of treatment (Fig. 1e); at later times (2–4 h), Sym004 was also detected within internal vesicles of late endosomes/multivesicular bodies (LEs/MVBs), as well as clustering on their limiting membranes (Supplementary Fig. S1b).

### Sym004 promotes EGFR localisation within a detergent-insoluble fraction

Since we observed Sym004 trafficking towards LEs/MVBs, we hypothesised that it promoted lysosomal degradation of EGFR. Surprisingly, neither lysosomal (bafilomycin A1) nor proteasomal (MG132) inhibitors prevented the decrease in EGFR levels at 2 h of Sym004 treatment (Fig. 1f). In contrast, EGF-mediated EGFR degradation was blocked by bafilomycin A1. We therefore hypothesised that the observed decrease in EGFR levels was not an actual degradation; instead, EGFR molecules cross-linked by Sym004 accumulated within a detergent-insoluble fraction (DIF) following centrifugation of cell lysates. To test this, we used three different lysis buffers: CellLytic M (buffer used so far; commercially available, containing a mild detergent), RIPA buffer (1% sodium deoxycholate) and Laemmli buffer (2% SDS). We found that cell lysis with a mild detergent (CellLytic M) led to EGFR accumulation within DIF upon treatment with Sym004, but not EGF (Fig. 1g). In the case of RIPA buffer, which is presumably more stringent, EGFR no longer accumulated within DIF; instead, we recovered more EGFR within cell lysates (Fig. 1g). Similarly, Laemmli buffer recovered more EGFR, although not as much as RIPA buffer. Note that there was no DIF in the case of Lammeli buffer, because the lysis protocol did not involve the centrifugation step. We observed a similar phenomenon of resistance to detergent extraction in two other HNC cell lines (Supplementary Fig. S1c).

We then addressed the question whether EGFR ‘trapped’ within DIF included the fraction of the receptor present at the plasma membrane. The cells were incubated with Sym004 on ice (4 °C) to prevent internalisation followed by lysis with a mild detergent (CellLytic M), or incubated for further 2 h at 37 °C before lysis. We found that EGFR already accumulated within DIF upon cell incubation on ice in the presence of Sym004, but not cetuximab or EGF; EGFR was also present within DIF upon incubation at 37 °C (Fig. 1h). These findings indicate that DIF included Sym004-bound EGFR present on the cell surface.

### Sym004 promotes lysosomal degradation of EGFR

**Figure 2:**
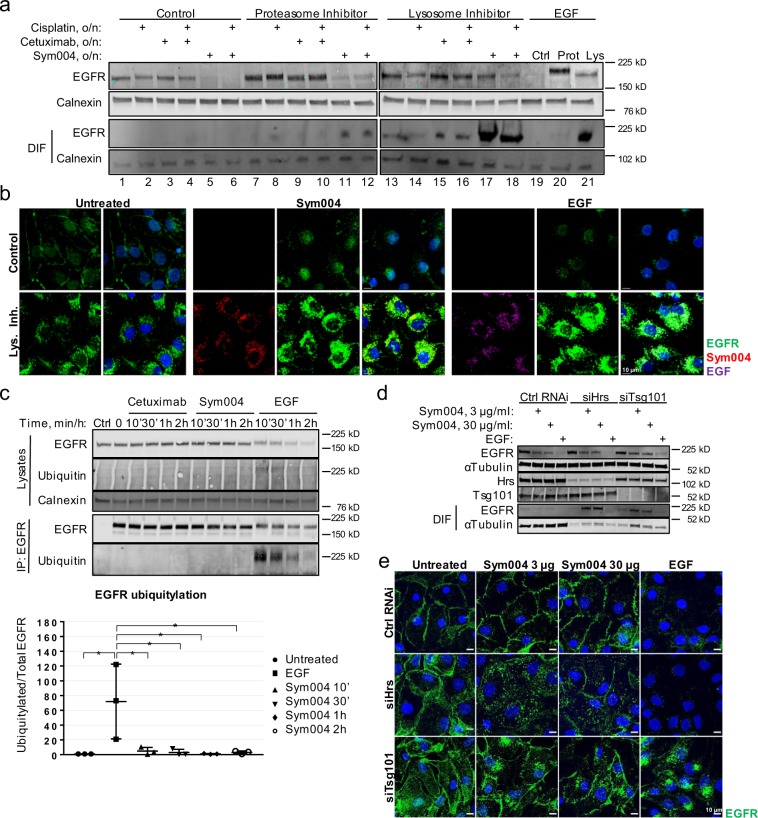
Sym004 promotes ubiquitin-independent, ESCRT-dependent lysosomal degradation of EGFR. **(a)** SCC47 cells were serum-starved for 1 h, then MG132 (10 µM) or bafilomycin A1 (600 nM) were added, followed 1 h later by cisplatin (50 µM), cetuximab, Sym004 or EGF. The cells were incubated overnight and lysed with RIPA buffer. **(b)** SCC47 cells were serum-starved for 1 h, then bafilomycin A1 (600 nM) was added followed 1 h later by Sym004-AF568 or EGF-AF647. The cells were incubated overnight, fixed and stained with EGFR-AF488 antibody. **(c)** Serum-starved SCC47 were treated with cetuximab, Sym004 or EGF as indicated, then lysed. EGFR was immunoprecipitated with anti-EGFR antibody (normal mouse IgG used in Control). For quantifications, ubiquitylated EGFR was normalised to total EGFR. **(d)** Serum-starved, Hrs- or Tsg101-depleted (or control) SCC47 cells were treated with Sym004 (3 µg/ml or 30 µg/ml) or EGF for 6 h, then lysed. **(e)** The cells treated as in (d) were fixed and stained with EGFR-AF488. Nuclei were stained with Hoechst 33342. All immunoblots were cropped for clarity.

We then analysed whether prolonged Sym004 exposure ultimately led to EGFR lysosomal degradation. The cells were treated overnight with Sym004, cetuximab, EGF or a chemotherapeutic drug cisplatin, in the presence or absence of the proteasomal or lysosomal inhibitors, and subsequently lysed with RIPA buffer (strong detergent). Using these conditions, we showed that Sym004 mediated lysosomal degradation of EGFR, because pre-treatment with bafilomycin A1, but not MG132, rescued EGFR levels (compare lines 11 and 17 in Fig. 2a). Although cisplatin has been shown to promote EGFR degradation in HNC cells^14^, its effect on EGFR level was minimal (line 1 and 2), thus indicating that Sym004 promoted EGFR degradation to a much greater extent than stress (cisplatin). Notably, proteasomal inhibition also rescued EGFR levels upon EGF, but not Sym004 (compare lines 11 and 20). It has been shown previously that inhibition of proteasomal function interferes with EGFR degradation due to depletion of the free ubiquitin pool within the cell^15^. Thus, our data suggest that although the ubiquitin moieties retrieved by functioning proteasome system are required for EGF-mediated degradation, they are dispensable for Sym004-mediated lysosomal degradation of EGFR.

Strikingly, when lysosomal function was impaired upon overnight treatment, large amounts of EGFR accumulated within DIF in the case of both Sym004 and EGF (see DIF in lines 17, 18 and 21 in Fig. 2a). Bafilomycin A1 prevents lysosomal acidification and inhibits fusion of LEs/MVBs with lysosomes, leading to the accumulation of cargo unable to undergo degradation^16,17^. It is likely, therefore, that the observed population of EGFR trapped within DIF represents the receptors accumulated within perinuclear LEs/MVBs, rather than on the cell surface (as in Fig. 1f). To confirm this, we took advantage of fluorescently-labelled Sym004 to allow for imaging of the fluorescent signal from within DIF without the risk of Sym004-EGFR complexes being inaccessible for immunostaining. Additionally, upon standard permeabilisation conditions, the cells were stained with fluorescent anti-EGFR antibody; this approach was used to verify whether our immunostaining protocol allowed the detection of EGFR trapped within DIF. As expected, in untreated control (DMSO) cells, EGFR predominantly localised to the plasma membrane, and upon inhibition of lysosomal function EGFR additionally accumulated within the perinuclear region; this indicated the existence of a basal level of EGFR trafficking (Fig. 2b). Treatment with fluorescently-labelled Sym004 or EGF resulted in potent EGFR degradation, whereas in the presence of bafilomycin A1, both fluorescent Sym004 and EGF accumulated within the perinuclear compartment; they also colocalised with anti-EGFR antibody thus verifying our immunostaining protocol. In summary, we showed that both Sym004 and EGFR underwent lysosomal degradation upon Sym004 exposure, and accumulated within perinuclear DIF when lysosomal function was impaired.

### Sym004-mediated EGFR degradation is ubiquitin-independent

Diverse subpopulations of endocytic vesicles have been described^18,19^ and we have shown previously that stress (UVC, cisplatin) and ligand induce EGFR accumulation within distinct subpopulations of LEs/MVBs^20^. Thus, we next analysed whether Sym004 and EGF traffic through the same, or distinct, pool(s) of endosomes. The cells were pre-treated with bafilomycin A1 to inhibit lysosomal degradation and allow for EGF/Sym004 accumulation. The cells were then stimulated with fluorescently-labelled EGF and Sym004 and the trafficking was imaged in live cells. Note that Sym004 competes with ligand binding, therefore EGF was added 5 min prior to Sym004 to allow the initial internalisation of the EGF:EGFR complexes. We found that Sym004 partially colocalised with EGF within perinuclear vesicles (Supplementary Fig. S2a) indicating that, unlike stressors, Sym004-internalised EGFR trafficks through the same population of LEs/MVBs as EGF-stimulated EGFR.

Previous studies have shown contrasting results on receptor ubiquitylation upon treatment with antibody mixtures^6,8^. Therefore we next analysed the EGFR ubiquitylation status upon various treatments. As expected, EGF triggered strong and rapid EGFR ubiquitylation, which gradually decreased over time (Fig. 2c). In contrast, EGFR ubiquitylation was undetectable upon treatment with Sym004 for up to several hours (Fig. 2c and Supplementary Fig. S2b). Importantly, the antibody used for the detection of EGFR ubiquitylation recognises all forms of ubiquitin chains^21^. Additionally in A431NS skin cancer cells, Sym004 did not promote phosphorylation of EGFR Y1045, which is the major binding site for Cbl ubiquitin ligases shown to be critical for EGFR ubiquitylation^22,23^ (Supplementary Fig. S2c). We further found that Sym004-mediated EGFR degradation was independent of Cbl ubiquitin ligases, because depletion of cCbl and Cbl-b partially rescued EGFR levels upon EGF stimulation, but had no effect on EGFR degradation upon Sym004 (Supplementary Fig. S2e).

We next addressed the question of whether the ESCRT machinery is required for Sym004-mediated degradation. We found that both the ESCRT-0 component Hrs and the ESCRT-I component Tsg101 contributed to this process, because depletion of either of them partially prevented Sym004-mediated EGFR degradation, which instead accumulated within DIF (Fig. 2d,e). We showed previously that an accessory protein Alix is required for sorting of non-ubiquitylated EGFR onto ILVs of MVBs upon exposure to stress (UVC), but not EGF^20^. Consistently, Alix was dispensable for EGF-mediated degradation of ubiquitylated EGFR (Supplementary Fig. S3a); however, it was also dispensable for Sym004-mediated degradation of non/negligibly-ubiquitylated EGFR.

### The EGFR interactome upon Sym004 resembles that upon EGF

**Figure 3:**
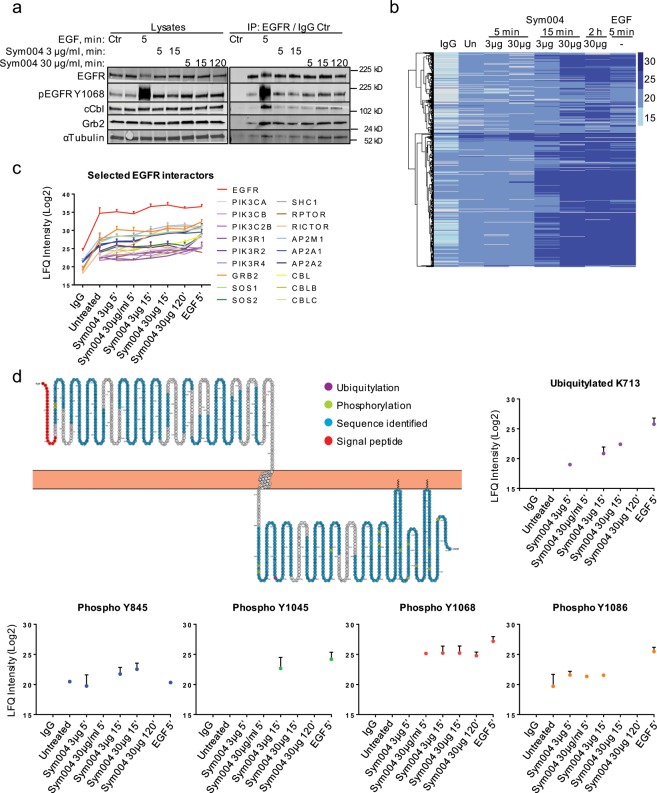
EGFR interactome upon Sym004 resembles that upon EGF, but differs in kinetics of binding with further differences in EGFR phosphorylation and ubiquitylation events. **(a)** Serum-starved SCC47 cells were treated with Sym004 (3 µg/ml or 30 µg/ml) for various times (5 min, 15 min or 120 min), or with EGF (50 ng/ml, 5 min), then lysed with mass spectrometric lysis buffer (see Methods). EGFR was immunoprecipitated using EGFR antibody previously cross-linked to beads (normal mouse IgG used in Control). **(b)** The cells from (a) were subjected to a mass spectrometric sample processing and analysis, and the LFQ protein intensities were subjected to hierarchical clustering analysis to produce a heatmap highlighting differentially abundant proteins in each condition. Darker shades correspond to a relatively high abundance while lighter shades correspond to a relatively low abundance. **(c)** Examples of selected EGFR interactors. **(d)** A schematic representation of the EGFR sequence generated using Protter^70^ showing sequence coverage, all identified phosphorylation and ubiquitylation modifications within EGFR and the signal peptide of 24 amino acids. These 24 amino acids were subsequently subtracted from the identified modifications for the final amino acid numbering. Selected modifications are represented on the graphs. Error bars, SD (if a protein was identified in at least two replicates). LFQ, label-free quantification. All immunoblots were cropped for clarity.

We next set out to analyse the EGFR interactome upon treatment with Sym004 or EGF. Following serum-starvation and stimulation with EGF (50 ng/ml, 5 min) or Sym004 (3 µg/ml or 30 µg/ml) for various times (5–120 min), EGFR was immunoprecipitated and the samples were first subjected to immunoblotting. We confirmed that EGF stimulation resulted in profound binding of both cCbl ubiquitin ligase and Grb2, an adaptor protein essential for MAPK/Erk signalling (Fig. 3a)^24^. Unexpectedly, we also found that they interacted with EGFR upon prolonged Sym004 exposure (15 min and 120 min), although to a lesser extent compared to EGF (Fig. 3a).

The samples were then subjected to a mass spectrometric analysis of the total EGFR interactome. Using this unbiased approach, we further unveiled similarities in EGFR interactors between short (5 min) EGF stimulation and prolonged (15 min and 120 min) Sym004 treatment (Fig. 3b). In particular, we showed that many interactors, such as Grb2, PIK3CA and the Cbl ubiquitin ligases, bound to EGFR in the presence of both EGF and Sym004 (Fig. 3c). This was surprising, because we observed profound differences between Sym004 and EGF in their effects on EGFR signalling and ubiquitylation (Fig. 2c, Supplementary Fig. S2c,d). Since we detected EGFR interaction with the Cbl ubiquitin ligases (Fig. 3a,c), we cannot exclude the possibility that EGFR ubiquitylation upon Sym004 treatment was below the detection limit of our immunoblotting technique. Alternatively, it might be that the observed interaction was non-functional and did not lead to efficient EGFR ubiquitylation. This possibility was supported by the observation that EGFR similarly interacted with several MAPK/Erk pathway components (Grb2, SOS1 and Shc), as well as with phosphoinositide 3-kinases, upon prolonged (120 min) treatment with Sym004 (Fig. 3c), but at this point, it inhibited downstream signalling (Supplementary Fig. S2c,d).

### Major differences in EGFR phosphorylation status between Sym004 and EGF

We then analysed the phosphorylation status of EGFR and found that Sym004 exposure resulted in persistent phosphorylation of EGFR on Y1068, although to a much lesser extent compared to EGF (Fig. 3d); this was consistent with our immunoblotting data (Supplementary Fig. S2c,d). In contrast, phosphorylation of Y1086, which was greatly enhanced in the presence of EGF, was inhibited upon prolonged Sym004 exposure (120 min). Similarly, phosphorylation of Y845, a tyrosine residue located within the EGFR activation loop, was blocked by prolonged Sym004 treatment. Finally, EGF stimulation resulted in strong phosphorylation of Y1045, which has been shown critical for efficient Cbl engagement^22^. In contrast, weak and transient (15 min) Y1045 phosphorylation in the presence of Sym004 was undetectable upon prolonged treatment (Fig. 3d and Supplementary Fig. S2c); additional phosphorylated residues identified within EGFR are presented in Supplementary Fig. S4. In summary, Sym004 triggered prolonged but modest EGFR Y1068 phosphorylation and only transient phosphorylation of other critical tyrosine residues.

These differences in EGFR phosphorylation status presumably reflect the different outcomes that Sym004 and EGF exert on downstream signalling. It is possible that EGFR Y1068 phosphorylation is sufficient for binding of many EGFR interactors, such as Grb2 or Cbl ligases; however, the absence of other phosphorylated residues (Y845, Y1045, Y1086) upon prolonged Sym004 exposure prevents these proteins from exerting their correct functions, *i.e*. Cbl from promoting EGFR ubiquitylation, and PI3Ks, Grb2, Shc and Sos from activation of downstream signalling. As a proof of principle, we identified a single EGFR ubiquitylation site (K713), one of the major sites described previously^25^ and found that low levels of EGFR ubiquitylation could be detected upon transient (5–15 min) Sym004 exposure, but were undetectable upon prolonged treatment (120 min); importantly, EGFR degradation was minimal at this point (Figs. 1e and 3a). Moreover, these low levels of ubiquitylation coincided with transient EGFR phosphorylation at Y845, Y1045 and Y1086 (Fig. 3d) and transient activation of downstream signalling (Supplementary Fig. S2c,d). These data suggest that upon treatment, Sym004 does not immediately exert its correct inhibitory functions, which likely commence only upon binding to a sufficient number of EGFR molecules at the plasma membrane, *i.e.* after the initial 5–15 min.

### Sym004 induces prolonged and asynchronous EGFR internalisation and degradation

**Figure 4:**
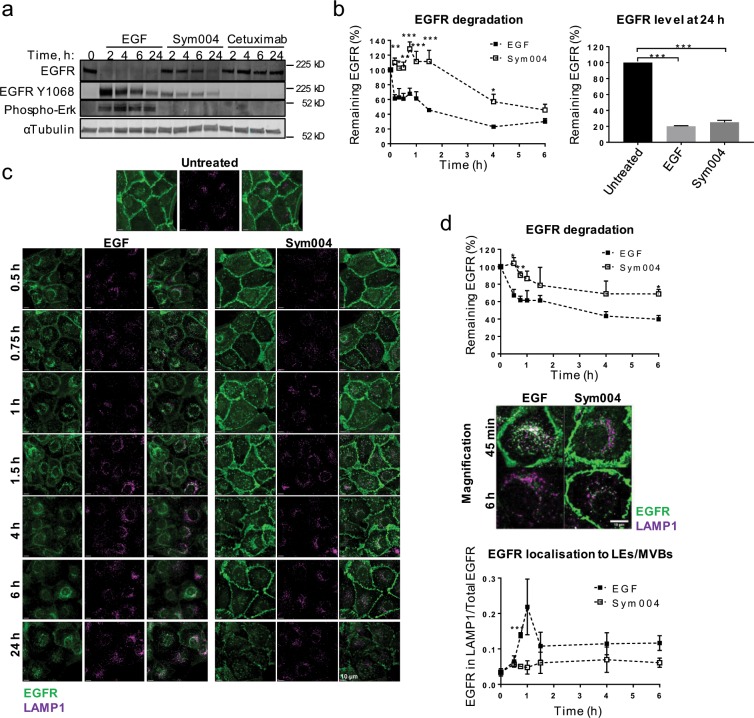
Sym004 induces asynchronous EGFR endocytosis and delayed degradation compared to EGF. **(a)** Serum-starved SCC47 cells were treated with EGF, Sym004 or cetuximab, then lysed. **(b)** Serum-starved SCC47 cells were treated with Sym004 or EGF for 0–24 h, then lysed. EGFR degradation was quantified as a percentage of remaining EGFR (normalised to calnexin) relative to untreated cells. **(c)** Serum-starved SCC47 cells were treated with Sym004 or EGF, fixed and stained with EGFR-AF488 and LAMP1-AF647. **(d)** Magnification of cells in (c). For quantification of EGFR degradation in (c), background fluorescence was subtracted from EGFR fluorescence, and the signal was normalised to untreated cells. For quantification of EGR delivery to late endosomes, EGFR signal within LAMP1 surface was normalised to total EGFR signal (see Methods). *p < 0.05, **p < 0.01, ***p < 0.001. Error bars, SEM. All immunoblots were cropped for clarity.

Although we found similarities in the EGFR interactome between Sym004 and EGF, there was a strong difference in the kinetics of binding (5 min for EGF and 15–120 min for Sym004) and in the kinetics of EGFR degradation (Figs. 3b and 4a). To investigate this in more detail, the cells were incubated for various times with either Sym004 or EGF, and EGFR degradation was quantified *via* immunoblotting. Note that the high dose of EGF (50 ng/ml) was expected to promote efficient EGFR ubiquitylation and degradation, as compared to recycling of non/negligibly-ubiquitylated EGFR upon low EGF doses (<3 ng/ml)^22,26^. We confirmed that strong EGFR degradation was already detected within 30 min of EGF stimulation (~61% EGFR remaining), with the majority of EGFR being degraded within 4 h (~23% EGFR remaining) (Fig. 4b). In contrast, Sym004-induced degradation proceeded at a much lower rate, could not be detected within the initial 90 min, and a large proportion of EGFR was still present at 4 h of treatment (~57%). Notably, both Sym004 and EGF ultimately led to similar levels of EGFR degradation at 24 h (~25% and 20% EGFR remaining, respectively) (Fig. 4b).

To confirm this, we then analysed EGFR degradation *via* immunofluorescence. The results similarly showed that Sym004-mediated degradation of EGFR was delayed compared to EGF-mediated degradation (Fig. 4c,d, top). Next, we analysed the kinetics of EGFR delivery to LEs/MVBs and found a sharp increase in EGFR colocalisation with LAMP1 (lysosome-associated membrane protein 1) within the first hour of EGF stimulation followed by a reduction in colocalisation, which correlated with EGFR degradation (Fig. 4d, bottom)^27^. In contrast, Sym004 treatment did not result in a coordinated EGFR delivery to LEs/MVBs; instead, EGFR trafficking was slow and gradual, and a large proportion of EGFR remained at the plasma membrane for several hours. We then addressed the possibility of EGFR recycling contributing to prolonged plasma membrane location, but found that neither depletion of Rab11 (Supplementary Fig. S3b,c) nor Vps35, a critical component of the retromer complex that promotes cargo recycling (Supplementary Fig. S3b,c)^28^, prevented EGFR accumulation on the cell surface, indicating that it did not rely on the classical (slow) recycling or sorting pathways.

### EGFR tyrosine kinase activity is dispensable for Sym004-mediated endocytosis

**Figure 5:**
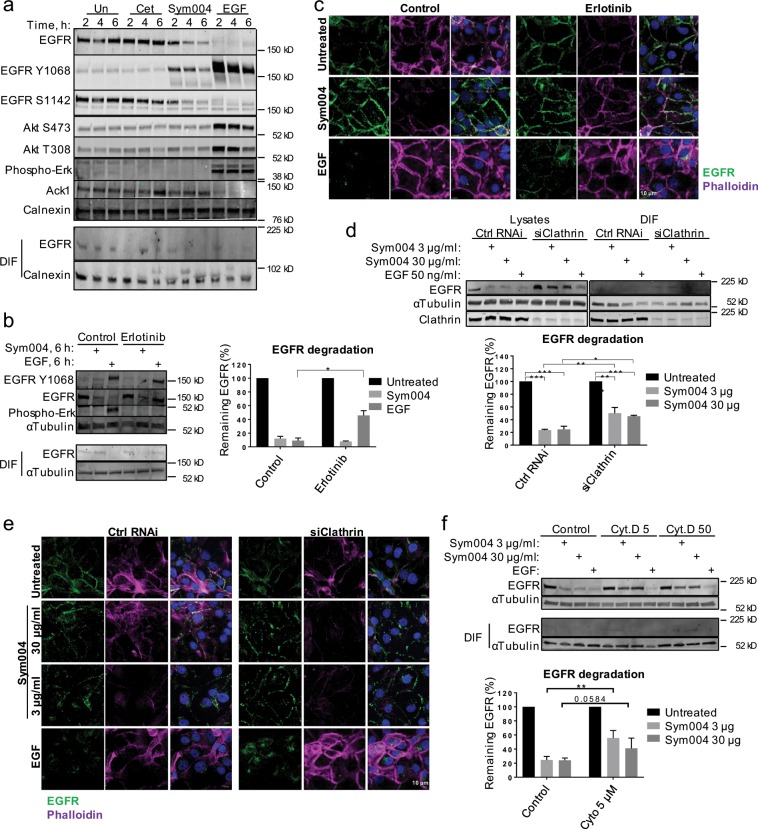
Sym004-mediated endocytosis does not require EGFR TK activity and only partially depends on clathrin. **(a)** Serum-starved SCC47 cells were treated with cetuximab, Sym004 or EGF, then lysed. **(b)** SCC47 cells were serum-starved, final 30 min in the presence of erlotinib (0.5 µM). The cells were treated with Sym004 or EGF for 6 h, then lysed. EGFR degradation was quantified as remaining EGFR (normalised to tubulin) relative to untreated cells. **(c)** The cells treated as in (b) were fixed and stained with EGFR-AF488 and Phalloidin-AF647 antibodies. **(d)** Serum-starved, clathrin heavy chain-depleted (or control) SCC47 cells were treated with Sym004 (3 µg/ml or 30 µg/ml) or EGF for 6 h, then lysed. EGFR degradation was quantified as remaining EGFR (normalised to tubulin) relative to untreated cells. (**e**) The cells treated as in (d) were fixed and stained with EGFR-AF488. **(f)** SCC47 cells were serum-starved, final 30 min in the presence of cytochalasin D (5 µM or 50 µM) or DMSO (control). The cells were treated with Sym004 or EGF for 6 h, then lysed. EGFR degradation was quantified as remaining EGFR (normalised to tubulin) relative to untreated cells. Error bars, SEM, *p < 0.05. Nuclei were stained with Hoechst 33342. All immunoblots were cropped for clarity.

Since we and others have shown that both Sym004 and HER2 antibody mixtures triggered subtle phosphorylation of their target receptors (Fig. 5a, Supplementary Fig. S2c,d)^8,29^, we next assessed whether EGFR tyrosine kinase (TK) activity was required for Sym004-mediated endocytosis. We found that small molecule inhibitor erlotinib partially prevented EGFR internalisation and degradation upon EGF, but had no effect on Sym004-mediated EGFR traffic (Fig. 5b,c). Interestingly, a non-receptor tyrosine kinase Ack1, which has been shown degraded alongside EGFR following EGF stimulation, was not affected by Sym004 treatment, indicating further differences between EGF- and Sym004-mediated traffic^30,31^ (Fig. 5a). We also showed that Sym004 promoted strong EGFR degradation in two other HNC cell lines, which similarly was not affected by erlotinib treatment (Supplementary Fig. S5a,b). Note that EGF treatment did not promote significant EGFR degradation in PCI30 and HN5 cells, which express much higher EGFR levels than SCC47 cells (Supplementary Fig. S5a,c), likely due to the EGF dose used (50 ng/ml) being insufficient to promote efficient EGFR degradation in these cells.

We have shown previously that stressors such as UVB, UVC and cisplatin require p38 activity to induce EGFR endocytosis^20,32^. Thus, we next analysed whether Sym004-mediated endocytosis required p38 activity. SCC47 cells were treated with p38 inhibitor and, following incubation with cetuximab, Sym004, EGF or cisplatin, cell surface proteins were biotinylated and subsequently purified. As expected, cisplatin promoted p38 activation, represented by phosphorylation of a p38 substrate HSP27, and p38 inhibitor greatly reduced this phosphorylation (Supplementary Fig. S5d). Additionally, inhibition of p38 activity partially rescued EGF-mediated EGFR degradation, likely through interference with Cbl-mediated ubiquitylation^33^; however, p38 inhibitor did not prevent Sym004-mediated EGFR removal from the cell surface.

### Sym004-mediated endocytosis partially depends on clathrin, dynamin activity and actin polymerisation

Previous studies have suggested various routes of endocytosis for cross-linked receptors, including CME, caveolar endocytosis and macropinocytosis^6–8,34^. Thus, we next depleted the cells of AP2α1, an adaptor protein involved in CME, and found that AP2α1 depletion had no effect on EGFR internalisation upon Sym004 (Supplementary Fig. S6a,b); however, AP2α1 depletion did not prevent internalisation of transferrin, indicating that it was insufficient to inhibit CME (Supplementary Fig. S6c). In contrast, depletion of clathrin heavy chain (CHC) completely blocked transferrin uptake (Supplementary Fig. S6c). Therefore, using this approach we next showed that only ~15–30% of EGFR was rescued from degradation by clathrin depletion (Fig. 5d), indicating that CME contributed to the uptake of a proportion, but not the majority of the EGFR-Sym004 complexes; note that EGFR did not accumulate within DIF, thus indicating genuine degradation (Fig. 5d). We further showed that EGFR degradation was independent of Sym004 concentrations, and a large proportion of EGFR (~45–60%) was degraded at both high and low Sym004 concentrations independently of clathrin depletion (Fig. 5d,e). This is in contrast to the uptake of EGF-stimulated EGFR, where CME has been shown to be the predominant internalisation pathway only upon low EGF doses, and has been coupled to EGFR recycling^35^; instead, upon Sym004 treatment, a similar proportion of EGFR is endocytosed through CME and subsequently degraded upon both high and low Sym004 doses.

Next, we assessed whether dynamin, a large GTPase which mediates vesicle scission during CME, caveolar endocytosis and other types of internalisation^36^, was required for Sym004-mediated endocytosis. We took advantage of a dominant-negative mutant dynamin-K44 (Lys44Ala) with impaired GTP binding, thus unable to perform vesicles scission^37^. We showed that internalisation of fluorescently-labelled Sym004 was marginally affected by dynamin-K44 expression; in contrast, internalisation of fluorescent EGF was nearly completely blocked in the presence of this mutant (Supplementary Fig. S7a).

Since we noticed that Sym004 treatment may have induced alteration in actin cytoskeleton (Fig. 5c,e), we next addressed the possibility that actin polymerisation was required for the uptake of the EGFR-Sym004 complexes and found that interfering with actin filament assembly partially rescued EGFR degradation (Fig. 5f and Supplementary Fig. S7b). Actin has been linked to both clathrin-dependent and -independent endocytosis, but also proposed to facilitate membrane scission during fluctuation force-mediated endocytosis of Shiga toxin^36,38^. The toxin binds to a cell surface lipid and has been found associated with detergent-resistant lipid rafts. Nonetheless, Sym004-mediated uptake did not require lipid raft formation, because EGFR degradation was unperturbed by depletion of either caveolin-1 or flotillin 1/2 (Supplementary Fig. S6a,b and d).

Finally, we investigated whether Sym004 uptake proceeded *via* macropinocytosis, which can be both dynamin-dependent and -independent^39^. The cells were treated with fluorescent Sym004 or EGF in combination with dextran, a cargo of macropinosomes. We found that already within 10 min of treatment, both dextran and EGF displayed intracellular accumulation and partial colocalisation. In contrast, Sym004 was predominantly localised to the cell surface, and the minimal intracellular signal did not overlap with dextran (Supplementary Fig. S7c); at 30 min of treatment, the vast majority of Sym004 still remained at the plasma membrane. Since macropinocytosis is described a fast route of endocytosis, even when compared to CME^39^, it is an unlikely route for slow and asynchronous uptake of Sym004. In summary, these data indicate that EGFR uptake upon Sym004 exposure does not rely on a specific endocytic machinery, but instead Sym004 exploits several mechanisms to enable the internalisation of the EGFR-Sym004 complexes.

### Sym004 is more potent in HNC cells with higher EGFR cell surface expression and lower ratio of endosomal EGFR

**Figure 6:**
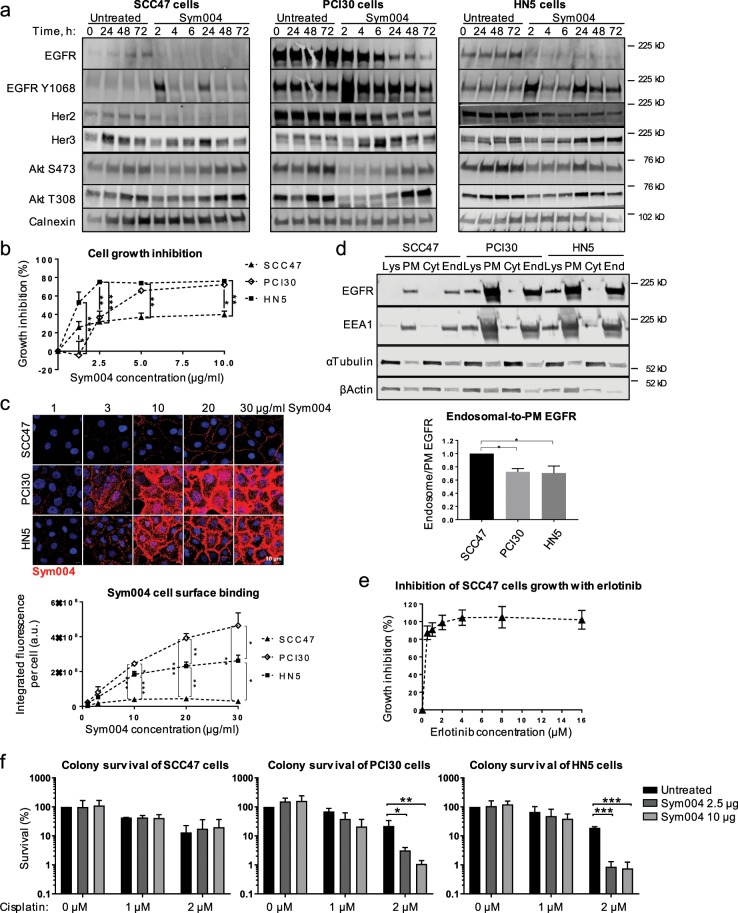
Sym004 potentiates cisplatin response in HNC cells with high Sym004 cell surface binding and lower endosomal-to-plasma membrane EGFR ratio. **(a)** Steady-state (not starved) SCC47, HN5 and PCI30 cells were either left untreated, or treated with Sym004 for indicated times, then lysed. **(b)** Steady-state cells were treated as indicated with Sym004 for 72 h, then fixed and stained with SRB to calculate cell growth inhibition (see Methods). **(c)** Serum-starved SCC47 cells were incubated with Sym004-AF568 on ice for 30 min, then fixed. Sym004-AF568 cell-surface binding was quantified (see Methods). **(d)** Subcellular fractionation was performed (see Methods). For quantifications, End EGFR was divided by PM EGFR, and normalised to the ratio in SCC47 cells. Lys, lysates; PM, plasma membrane; Cyt, cytosol; End, endosome. **(e)** Steady-state cells were treated as indicated with erlotinib for 96 h, then fixed and stained with SRB to calculate cell growth inhibition (see Methods). **(f)** Colony formation assay was performed (see Methods). The colonies formed were counted and adjusted for plating density. The colony survival was quantified as adjusted number of colonies normalised to untreated cells. Error bars, SEM, *p < 0.05, **p < 0.01, ***p < 0.001. Nuclei were stained with Hoechst 33342. All immunoblots were cropped for clarity.

We confirmed in three HNC cell lines that Sym004 promoted strong EGFR degradation and, when added simultaneously with EGF, Sym004 prevented activation of downstream signalling (Supplementary Fig. S8a). We then treated the cells for up to 72 h with Sym004 to analyse the effects of this prolonger treatment on cell signalling. Importantly, no mutations within the *Ras*/*Raf* oncogenes, which could have led to the resistance to EGFR-targeted therapy, have been reported in either of these cell lines^40^. We found that all three cell lines responded in a similar manner to Sym004 treatment, with an initial increase in EGFR phosphorylation (at 2 h) that decreased concomitantly with EGFR degradation (Fig. 6a). At 24 h, there was another peak in EGFR phosphorylation that correlated with upregulation of HER3 and re-activation of downstream signalling; HER2 was degraded alongside EGFR, presumably due to hetero-dimerisation. Notably, HER3 expression was more pronounced in PCI30 and HN5 cells (both with high EGFR levels); this may reflect more potent EGFR degradation in these cells, which would be compensated by higher HER3 expression. Altogether, these data are consistent with previous reports showing that EGFR-targeting antibodies may promote upregulation of HER2/HER3 expression, leading to re-activation of downstream signalling^41^.

We next analysed the effects of prolonged (72 h) Sym004 exposure on inhibition of cell proliferation and found profound differences between the three cell lines: Sym004 exposure resulted in only ~40% inhibition of the proliferation of SCC47 cells, whereas the inhibition was much more efficient in PCI30 and HN5 cells (~70–75%) (Fig. 6b).

We then analysed the ability of these HNC cells to bind Sym004 at the plasma membrane, as an indicator of EGFR cell surface expression. The cells were placed on ice to prevent EGFR internalisation, and incubated with various concentrations of fluorescently-labelled Sym004. We found that several fold more Sym004 bound to the surface of PCI30 cells (10–16 fold) and HN5 cells (6–10 fold) than to SCC47 cells (Fig. 6c). Importantly, in all cell lines we approached the saturating concentrations of Sym004, because at higher doses, an increase in concentration did not lead to any further increase in binding (Supplementary Fig. S8b). These differences indicate that PCI30 and HN5 cells expressed significantly more EGFR on the cell surface.

Although EGFR predominantly resides at the plasma membrane, it undergoes continuous trafficking through the endocytic system, from where it can send proliferative signals^42^ and we showed here the existance of a basal level of EGFR trafficking in SCC47 cells (Fig. 2b). We have also shown previously that in the case of stressors, active EGFR is retained within LEs/MVBs from where it continues to signal^20^. We therefore hypothesised that targeting of EGFR at the plasma membrane may be insufficient to promote significant cell death due to a higher proportion of endosomal EGFR unavailable for binding. Thus, we next analysed the ratio of endosomal-to-plasma membrane EGFR within the three HNC cell lines and found that in steady-state (not starved) PCI30 and HN5 cells, the endosomal-to-plasma membrane ratio of EGFR was ~30% lower compared to SCC47 cells (Fig. 6d). This endosomal EGFR would be inaccessible for Sym004 binding, rendering the cells less sensitive to EGFR-targeted mAbs. This is consistent with our data showing that Sym004 was much more potent in inhibiting cell proliferation of PCI30 and HN5 cells (Fig. 6b), both with lower endosomal-to-plasma membrane EGFR ratio (Fig. 6d). To confirm this, we treated SCC47 cells with EGFR TK inhibitor erlotinib that does not require EGFR cell surface expression and found that it potently inhibited cell proliferation (Fig. 6e), thus indicating that EGFR signalling is required for SCC47cell growth and reinforcing our hypothesis that EGFR accessibility on the plasma membrane is critical for the inhibitory function of Sym004.

Finally, we compared the three cell lines in their ability to form colonies upon combination of Sym004 and chemotherapy (cisplatin). We found that pre-treatment with Sym004 potentiated cytotoxic effects of cisplatin in PCI30 and HN5 cells, both with lower endosomal-to-plasma membrane EGFR ratio (Fig. 6d,f and Supplementary Fig. S8c). Note that 24 h of Sym004 treatment followed by a recovery in a drug-free medium (without cisplatin) did not promote cell death, likely because the cells were able to recoveir their EGFR levels. Interestingly, we only observed potentiation with an increasing dose of cisplatin (2 µM) and not with a lower dose (1 µM). This is consistent with previous work showing that Sym004 potentiation of radiation response is more prevalent with increasing doses of radiation^29^ and indicates that sufficient DNA damage may be required for EGFR-targeted therapy to have a potentiating effect. In conclusion, the combination of cisplatin and Sym004 was beneficial in HNC cells with higher EGFR plasma membrane expression and/or lower ratio of endosomal EGFR.

## Discussion

Antibody combinations are an innovative approach across various solid tumours and haematological malignancies^1,2^. The dual epitope targeting antibody combination Sym004 has been employed in several clinical trials with promising results^10,43,44^. To fully employ the benefits of such antibody combinations, it is critical to understand the biology behind the trafficking and removal of their target receptors.

**Figure 7:**
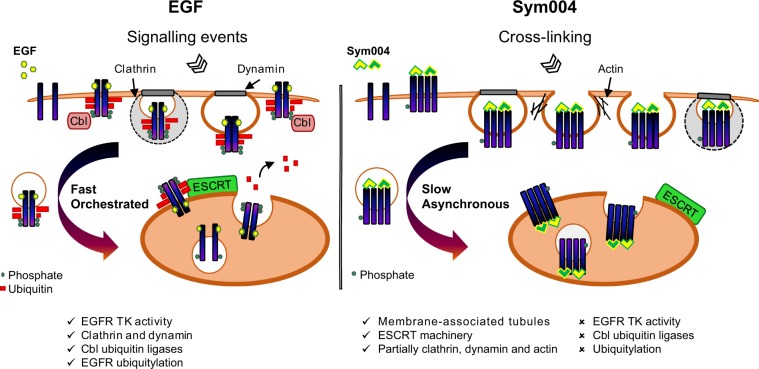
Proposed model of trafficking of EGFR cross-linked by Sym004. EGF promotes strong EGFR phosphorylation and Cbl-dependent ubiquitylation leading to synchronised, dynamin-dependent and clathrin-dependent and -independent internalisation, followed by fast and coordinated delivery to LEs/MVBs and ESCRT-dependent lysosomal degradation; both EGFR TK activity and p38 activity contribute to this process. Sym004-mediated EGFR internalisation is slow and asynchronous, partially depends on clathrin, dynamin activity and actin polymerisation, but does not require EGFR TK activity, p38 activity, Cbl ubiquitin ligases or EGFR ubiquitylation. Although the ESCRT machinery is required for EGFR degradation upon Sym004, it is likely important for LEs/MVBs formation *per se* rather than for recognition of non/negligibly-ubiquitylated EGFR. We propose that cross-linking of multiple EGFR molecules by Sym004 physically triggers EGFR endocytosis, leading to lysosomal degradation of both EGFR and Sym004. TK, tyrosine kinase; PM, plasma membrane, Les/MVBs, late endosomes/multivesicular bodies.

We set out to characterise the precise mechanism of EGFR endocytosis and degradation upon exposure to Sym004, and found that EGFR uptake promoted by Sym004 differed substantially from ligand-stimulated uptake (Fig. 7). In particular, low doses of EGF have been linked to clathrin-dependent EGFR endocytosis and its recycling to the plasma membrane, whereas saturation of the clathrin machinery upon high EGF concentrations (>3 ng/ml) has been shown to result in clathrin-independent uptake that also correlated with rapid EGFR ubiquitylation and degradation; preventing this ubiquitylation led to inefficient EGFR removal^25,26^ Our study uncovered that these well-described mechanisms of endocytosis and degradation of ligand-stimulated EGFR are not applicable to the antibody combinations.

Firstly, clathrin-mediated endocytosis contributed to the uptake of a proportion of EGFR independently of Sym004 concentrations, and the majority of EGFR was degraded *via* clathrin-independent mechanisms even at low Sym004 doses (Fig. 5d). Secondly, EGFR was efficiently degraded upon both high and low Sym004 concentrations (Figs. 2d, 5d, f, 6c). One possible explanation for this phenomenon would be that the cross-linking of a number of EGF receptors on the cell surface results in a non-specific uptake of EGFRs that are in a close proximity, or indeed that dimerise with the cross-linked receptors.

The EGFR TK activity, which was required for the uptake of ligand-stimulated EGFR (Fig. 5b,c), was dispensable for EGFR endocytosis upon Sym004. Additionally, EGFR uptake was greatly delayed compared to EGF, and the majority of the receptor remained at the plasma membrane for several hours. These Sym004-bound cell surface EGF receptors clustered within detergent-insoluble tubules, which were very distinct from the discrete puncta formed in the presence of EGF (Fig. 1c–e).

Strikingly, even at high Sym004 concentrations, we did not detect evident EGFR ubiquitylation (Fig. 2c and Supplementary Fig. S2b). In the canonical lysosomal degradation, ubiquitylation acts as a discrete signal for engagement of the ESCRT machinery^45,46^ and a non-ubiquitylated EGFR mutant has been found unable to undergo degradation^25^. Nonetheless, Sym004-bound EGFR underwent lysosomal degradation in the absence of ubiquitylation. Although we found that both Hrs and Tsg101 were required for Sym004-mediated EGFR degradation, this was unlikely to depend on the recognition of non/negligibly-ubiquitylated EGFR (Figs. 2c and 3d). We have shown previously that Hrs and Tsg101 have distinct roles during endosomal maturation: Tsg101 promotes LE/MVB formation and Hrs regulates accumulation of intraluminal vesicles^27^. Therefore, it is likely that Hrs and Tsg101 are required for MVB formation *per se* rather than for binding of non/negligibly-ubiquitylated EGFR upon Sym004 treatment.

In recent years, passive physical mechanisms have begun to emerge as important regulators of membrane bending during endocytic uptake. These include insertion of asymmetric proteins, lipid compaction and protein crowding and can be distinguished from the active mechanisms which rely on enzymatic activities or cytoskeletal reorganisation^47^; for example, steric interactions between crowded proteins may result in membrane bending, leading to the formation of elongated membrane-associated tubules. Since we showed unequivocally that Sym004 binding resulted in EGFR clustering within detergent-insoluble tubules, which underwent prolonged and asynchronous endocytosis over several hours, the EGFR uptake is unlikely to proceed through the orchestrated, well-described canonical uptake mechanisms. Instead, we propose that the physical forces exerted at the plasma membrane due to cross-linking of EGFR molecules initiate the internalisation of the EGFR-Sym004 complexes, and that the cross-linking is the critical factor in inducing membrane curvature, because the individual mAb cetuximab did not promote detectable EGFR internalisation.

The uptake of Shiga toxin, a protein produced by some bacterial strains of Shigella and Escherichia, has been shown to partially depend on clathrin, but also to be driven by membrane-mediated clustering forces exerted at the plasma membrane^38,48^. The toxin binds up to 15 molecules of the glycosphingolipid receptor (Gb3) and this tight binding restricts membrane fluctuations^49^. It has been proposed that fluctuation-induced force promotes toxin clustering, leading to the formation of tubular membrane invaginations^48^ and that dynamin, actin and endophilin-2A all contribute to the scission of Shiga toxin-induced tubules^38,50^. The possibility that a similar mechanism is involved in the antibody mixture-mediated endocytosis comes from the following observations: (1) both Sym004 and Shiga toxin lead to the formation of clusters at the plasma membrane; (2) both promote generation of tubular membrane invaginations; (3) both are accumulating within detergent-insoluble fractions; (4) internalisation of both Sym004 and Shiga toxin only partially depends on clathrin and dynamin activity; and (5) actin contributes to the uptake of both Shiga toxin and Sym004 (Fig. 5d–f and Supplementary Fig. S7a)^9,38,49,51^.

In the case of Shiga toxin, its toxicity depends on Gb3 receptor expression at the cell surface, and cells without Gb3 receptor are insensitive to Shiga toxin^52^. We propose that in HNC cells (without mutations that would render them insensitive to EGFR-targeted therapy, *e.g*. within *Ras*/*Raf* oncogenes), the response to EGFR-targeting antibodies similarly correlates with EGFR accessibility at the cell surface. In support, we showed that two HNC cell lines with high EGFR membrane expression and Sym004 binding responded more potently to EGFR inhibition compared to SCC47 cells with low EGFR membrane expression (Fig. 6 and Supplementary Fig. S5c). Although we cannot exclude the possibility that this was a cell type-specific effect dependent on another signalling pathway, we showed that the proliferation of SCC47 cells was blocked by EGFR TK inhibitor erlotinib that does not require EGFR presence on the cell surface (Fig. 6e). These data also argue against the possibility that total EGFR levels would be a predictor of the response to EGFR-targeted therapy, since SCC47 cells with very low EGFR expression greatly responded to EGFR TKi. These data further argue that unperturbed EGFR signalling is indispensable for SCC47 cell growth, and that the limited inhibitory effect of Sym004 in these cells was due to the reduced availability of EGFR molecules on the cell surface, rather than EGFR signalling being compensated by another signalling pathway.

In summary, it is clear that the precise mechanistic understanding of the biology behind the therapeutic antibody combinations is a critical factor in optimising cancer treatment. We showed here that targeting of EGFR with a combination of antibodies against non-overlapping epitopes in head and neck cancer cells resulted in unconventional receptor uptake that was independent of canonical signalling events. The possibility that such unconventional uptake mechanisms may be applicable to antibody combinations targeting other cell surface RTKs warrants further mechanistic studies.

## Methods

### Cell culture and treatment

Head and neck cancer cells UM-SCC47 (SCC47) and PCI30 cells were kindly provided by Dr Tim Fenton (School of Biosciences, University of Kent), and HN5 cells by Professor Kevin Harrington (Section of Cell and Molecular Biology, Institute of Cancer Research). These cell lines’ authentication and mycoplasma testing were both completed by Eurofins Genomics, and they were mycoplasma-negative. The cells were cultured in DMEM (Sigma-Aldrich) with 10% FBS (Thermo Fisher Scientific), 1% Penicillin/Streptomycin (Sigma-Aldrich) and 1% L-glutamine (Sigma-Aldrich) at 37 °C in 5% CO_2_. The cells were serum-starved overnight (unless otherwise stated), in the same medium without FBS. Human retinal pigment epithelium ARPE-19 cells stably expressing clathrin light chain fused to eGFP (herein referred to as GFP-CLC cells) were generated as previously described^13^. Cells were maintained in DMEM/F12 media supplemented with 10% FBS, 100 U/mL penicillin and 100 μg/mL streptomycin at 37 °C and 5% CO_2_. Prior to experiments, GFP-CLC cells were serum starved for a minimum of 2 h in DMEM/F12 media without supplements, and live-cell imaging was performed in serum free DMEM/F12 media without supplements or phenol red. A431NS were obtained from American Tissue Culture Collection (ATTC, USA) and regularly authenticated by LGC Standards, and the cells were mycoplasma negative. They were cultured in DMEM with 4 mM L-glutamine and 10% FBS at 37 °C in 5% CO_2_. Prior to the experiment, the cells were serum-starved overnight in 0.5% FBS.

### Reagents and antibodies

Sym004 (5 mg/ml) (used at 30 µg/ml, unless otherwise stated) was provided by Symphogen (Ballerup, Denmark). Cetuximab (Erbitux; 5 mg/ml) (used at 30 µg/ml, unless otherwise stated) was obtained from Merck-Serono (Darmstadt, Germany). Cisplatin (1 mg/ml) was obtained from Accord Healthcare, Harrow, UK. Other reagents included: Human EGF (E9644, Sigma-Aldrich, used at 50 ng/ml, unless otherwise stated), EGF Biotinylated, AF647-conjugated (E35351, Thermo Fisher Scientific), nProtein A Sepharose 4 Fast Flow (GE17–5280–01, GE Healthcare), Fluorescein-conjugated Dextran 70 kDa (D1822, Thermo Fisher Scientific), Hoechst 33342 (H3570, Thermo Fisher Scientific), plasmids wt dynamin 1 pEGFP (Addgene #34680) and K44A Dynamin 1 pEGFP (Addgene #34681) were generated by Sandra Schmid (UT Southwestern Medical Center, TX, USA), Lipofectamine 3000 (L3000008, Thermo Fisher Scientific), Lipofectamine RNAiMAX (13778075, Thermo Fisher Scientific), p38 inhibitor SB203580 (559389, Merck, 10 µM, 30 min pre-treatment), Erlotinib (CDS022564, Sigma-Aldrich, 0.5 µM, 30 min pre-treatment, unless otherwise stated), Bafilomycin A1 (196000, Merck, 600 nM, 1 h or overnight, as indicated), MG132 (474791, Merck, 10 µM), Cytochalasin D (504776, Merck, 5 µM or 50 µM, 30 min pre-treatment unless otherwise stated), RIPA buffer (9806 S, Cell Signalling Technology, used throughout, unless otherwise stated), CellLytic M (C2978, Sigma-Aldrich), Laemmli (0.0625 M Tris/HCl pH 6.8, 2% SDS, 10% glycerol). Protease (cOmplete ULTRA Tablets, EDTA-free, Roche) and phosphatase (PhosSTOP, Roche) inhibitors were added to each buffer prior to cell lysis.

Antibodies used for immunoblotting: mouse EGFR (2239, 1F4, Cell Signalling Technology), rabbit EGFR (4267, D38B1, Cell Signalling Technology; used only in Fig. 4b), rabbit phospho-EGFR (Y1068) (2234, Cell Signalling Technology), rabbit phospho-EGFR (Y845) (2231, Cell Signalling technology), rabbit phospho-EGFR (S1142) (ab76194, Abcam), rabbit phospho-EGFR (Y1045) (2237, Cell Signalling Technology), rabbit phospho-EGFR (T699) (3056, Cell Signalling Technology), mouse ubiquitin (P4D1, sc-8017, Santa Cruz Biotechnology), rabbit phospho-Akt (T308) (2965, C31E5E, Cell Signalling Technology), rabbit phospho-Akt (S473) (9271, Cell Signalling Technology), mouse Akt (2920, pan 40D4, Cell Signalling Technology), mouse phospho-Erk (sc-136521, Santa Cruz Biotechnology), rabbit cCbl (2179, 49H8, Cell Signalling technology), rabbit Cbl-b (9498, Cell Signalling Technology), rabbit Hrs (15087, D7T5N, Cell Signalling Technology), goat Tsg101 (sc-6037, M-19, Santa Cruz Biotechnology), rabbit ALIX (00013156, Covalab), phospho-HSP27 (S82) (9709, D1H2F6 XP, Cell Signalling Technology), rabbit Clathrin Heavy Chain (4796, D3C6 XP, Cell Signalling Technology), mouse α-Adaptin 1/2 (sc-17771, C-8, Santa Cruz Biotechnology), rabbit Caveolin-1 (3267, D46G3, Cell Signalling Technology), mouse Ack1 (sc-28336, A-11, Santa Cruz Biotechnology), rabbit calnexin (2433, Cell Signalling Technology), mouse α-tubulin (T5168, Sigma-Aldrich). Secondary donkey-anti mouse (926–68072, IRDye 680RD), donkey anti-rabbit (926–32213, IRDye 800CV) and donkey anti-goat (925–32214, IRDye 800CW) antibodies were from Li-Cor. Mouse EGFR (sc-120, 528, Santa Cruz Biotechnology) and normal mouse IgG (sc-2025, Santa Cruz Biotechnology) were used for immunoprecipitation.

Antibodies/reagents used for immunofluorescence: rabbit EGFR-AF488 (5616, D38B1 XP, Cell Signalling Technology), rabbit EGFR-AF594 (8742, D38B1 XP, Cell Signalling Technology), Alexa Fluor 633 Phalloidin (A22284, Thermo fisher Scientific), rabbit LAMP1 (D2D11 XP, 9091, Cell Signalling Technology; conjugated to AF647 in-house), rabbit phospho-Histone H2A.X (S139)-AF647 (9720, 20E3, Cell Signalling Technology). LysoTracker Red (L7528, DND-99, Thermo Fisher Scientific, used at 100 nM, 2 h pretreatment). Sym004, cetuximab and LAMP1 antibody were labelled using antibody labelling kit Alexa Fluor 568 (A20184) and 488 (A20181) from Thermo Fisher Scientific according to the manufacturer’s instructions. If required, buffer exchange using Amicon Ultra 0.5 ml Centrifugal Filters 100 K (UFC510024, Merck Millipore) was performed prior to labelling for optimal labelling conditions.

### cDNA transfection and RNA interference

SCC47 cells were transfected with plasmid DNA using Lipofectamine 3000 (Thermo fisher Scientific), according to manufacturer’s instruction, and the cells were analysed 48 h post-transfection. SCC47 cells were reverse-transfected using Lipofectamine RNAiMAX (Thermo Fisher Scientific), according to manufacturer’s instructions. Briefly, the siRNA-Lipofectamine complexes were prepared in a well, and the cells were added directly to them. Two rounds of silencing were performed 72 h apart, in order to improve knockdown efficiency. 24 h after the second round of transfection, the cells were serum-starved overnight and treated the following morning. All of the following siRNAs were the Human ON-TARGETplus siRNA SMART pools from Dharmacon: CLTC (1213) (L-004001-01-0005), AP2A1 (160) (L-012492-00-0005), CAV1 (857) (L-003467-00-0005), PDCD6IP (Alix) (L-004233-00-0005), CBLB (868) (L-003004-00-0005, Dharmacon), CBL (867) (L-003003-00-0005, Dharmacon), VPS35 (55737) (L-010894-00-0005), RAB11A (8766) (L-004726-00-0005), Flot1 (L-010636-00-0005), Flot2 (L-003666-01-0005) and Non-Targeting Pool (D-001810-10-20). The siRNA sequences for Hrs and Tsg101^27^, and Alix^53^ have been described previously.

### Immunoblotting

The cells were lysed with RIPA buffer (Cell Signalling Technology), unless otherwise stated. The other buffers used, when indicated, were CellLytic M (Sigma-Aldrich) and Laemelli (made in-house). Protein concentration was assessed using DC Protein Assay (5000111, BioRad) and equal amount of protein was dissolved using NuPAGE LDS Sample buffer 4X (NP0007, Thermo Fisher Scientific) and NuPAGE Sample Reducing Agent 10X (NP0009, Thermo Fisher Scientific), then boiled 5 min at 95 °C. The lysates were separated on Mini-PROTEON TGX or Criterion TGX Precast gels (BioRad) and the proteins were transferred onto nitrocellulose membranes using Trans-Blot Turbo Transfer System (BioRad). The membranes were blocked with Odyssey Blocking Buffer (TBS) (Li-Cor) and probed subsequently with primary and secondary antibodies. The immunoblots were analysed using Odyssey Imaging System (Li-Cor) and Image Studio Lite 5.2 software (Li-Cor). All images presented were cropped for clarity. The uncropped images of immunoblots presented within Figs. 1–6 are shown in Supplementary Fig. S9.

### Cell surface protein biotinylation assay

For cell-surface biotinylation assay, Pierce Cell Surface Protein Isolation Kit (89881, Thermo Fisher Scientific) was used according to manufacturer’s instruction with modifications. Briefly, the cells were incubated twice for 30 min at 4 °C with biotin solution, followed by quenching. The cells were lysed with RIPA buffer (Cell Signalling Technology), and the lysates adjusted for protein concentration. Following incubation with NeutrAvidin beads, the proteins were eluted by boiling 5 min at 95 °C with NuPAGE LDS Sample buffer 4X (NP0007, Thermo Fisher Scientific) and NuPAGE Sample Reducing Agent 10X (NP0009, Thermo Fisher Scientific).

### Confocal microscopy

Immunofluorescence, Live-cell imaging, Image analysis

#### Immunoflurescence

Cells were fixed with 4% paraformaldehyde (PFA) (43368.9 M, Alfa Aesar) and permeabilised for 3 min with ice-cold 0.2% Triton X-100 (Sigma-Aldrich), or fixed and permeabilised with −20 °C methanol (for LAMP1 staining only). Next, the cells were blocked with PBS containing 10% FBS and 5% BSA, and incubated with antibodies. For antibody labelling, the Antibody Labelling kit from Life Technologies was used. If necessary, prior to labelling, the antibody’s buffer was exchanged for PBS using Amicon Ultra-0.5 ml Centrifugal Filters 100 K (Millipore). For Sym004-AF568 cell-surface binding, the cells were serum-starved overnight, then incubated on ice for 30 min with Sym004-AF568, before fixing.

#### Live-cell imaging

The cells were washed in pre-warmed PBS before adding cell-imaging medium (sterile-filtered solution of 9.7 g/l Hanks Balanced Salt without phenol red and sodium bicarbonate (Sigma), 10 mM HEPES (Fisher Scientific), pH 7.4). The cells were imaged at 37 °C as indicated in Fig. legends. Images were acquired on a Carl Zeiss LSM880 inverted laser scanning microscope using an Plan-Apochromatic 63X/1.4NA oil immersion objective. 405 nm, 488 nm, 561 nm and 633 nm laser lines were used for excitation of Hoechst 33342, AF488/FITC, AF568/Texas Red and AF633/AF647 fluorophores, respectively. The images were acquired using a PMT detector or a 32-channel spectral GaAsP detector.

#### Image analysis

All analyses of confocal microscopy images/time lapses were performed using Imaris 9.2 software (Bitplane). All quantifications were performed from at least three independent experiments, with at least three images per every condition. For quantification of Sym004-AF568 cell-surface binding, the background fluorescence (untreated sample) was subtracted from the sum (integrated) fluorescence, and the fluorescence per cell was calculated by dividing this value by the number of nuclei per image. For quantification of EGFR degradation, the background fluorescence (unstained cells) was subtracted from the sum EGFR fluorescence, and the EGFR signal was normalised to Untreated cells. For EGFR-LAMP1 colocalisation, the LAMP1 surface was created that contained total LAMP1 signal; this surface was absent in the negative control (unstained cells). The background fluorescence (from unstained cells) was subtracted from the sum EGFR fluorescence, and the EGFR signal within the LAMP1 surface was divided by total EGFR signal.

#### Total internal reflection fluorescence microscopy (TIRFM)

Dual colour live-cell imaging of GFP-RPE cells was performed as previously described^54^. Briefly, cells were pre-treated for 5 min with Sym004-AF568 (10 μg/mL), followed by washes in PBS. Cells were then placed in DMEM/F12 medium without phenol red on a live cell imaging stage maintained at 37 °C and 5% CO_2_. Some cells were instead treated with EGF-Cy3b (10 ng/ml) during imaging as above, as indicated. Immediately after placing the cells on the live-cell imaging stage, time-lapse images were acquired at a framerate of 1 s and exposure times between 50–100 ms for red and green channels. Each time-lapse image series consisted of 500 total frames (250 frames/channel) acquired over approximately 5 min. For each condition, 5 total time-matched image series were obtained over 40 min. Images were acquired with a Quorum Diskovery TIRF module fitted onto a Leica DMi8 microscope equipped with a 63X/1.49 NA TIRF objective with a 1.8X camera relay (total magnification 108X), using 488 nm and 561 nm laser illumination, 527/30 and 630/75 emission filters and acquired using an Andor iXon EMCCD camera (Andor). The extent of co-localization of fluorescently-labelled EGF or Sym004 with eGFP-clathrin was determined by Pearson’s coefficient using Just Another Colocalization Plugin^55^ for Image J^56^ as previously described^57^. Briefly, the first pair of images (Sym004/EGF and clathrin channel pairs) of each timelapse was subjected to detection of Pearson’s coefficient of colocalization. The data come from 23 individual data points (cells) in the case of EGF, and 73 in the case of Sym004. The data are presented as Tukey box plot with whiskers.

### Electron microscopy

Colloidal gold sols (British Biocell International) were coupled to Sym004 at pH 9.3, followed by secondary stabilisation with 1% BSA as described^58^. SCC47 cells plated on glass coverslips were serum-starved and treated with Sym004-Gold 5 nm or 10 nm, in the presence of 0.2% BSA, for indicated times. The cells were then washed twice with ice-cold PBS and fixed with 2% paraformaldehyde/2% glutaraldehyde in 0.1 M cacodylate for 30 min at RT, then washed with PBS. Cells were then osmicated, treated with tannic acid, dehydrated, infiltrated with Epon and mounted on Epon stubs all as described^59^. After polymerisation overnight at 60 °C coverslips were removed from Epon stubs with liquid nitrogen. 70 nm sections were cut en face and stained with lead citrate before examination on a Jeol 1010 transmission electron microscope and images acquired with a Gatan OriusSC100B charged couple device camera.

### Mass spectrometric analysis of EGFR interactome

Antibody cross-linking, Immunoprecipitation, In-gel digestion, LC-MS/MS, Protein and phosphosite data analysis.

### Antibody cross-linking

For cross-linking^60^, Protein A-Sepharose 4B beads (P9424, Merck Millipore) were re-suspended in a column in 50 mM Tris pH 7 to make a 50% slurry, and incubated with EGFR antibody 528 (sc-120, Santa Cruz Biotechnology) or normal mouse IgG (sc-2025, Santa Cruz Biotechnology) overnight at 4 °C. The beads were then washed 3× with 0.2 M sodium borate pH 9, and incubated with a freshly made solution of 0.2 mM dimethyl pimelimidate in sodium borate for 40 min at RT. The beads were washed once with 0.2 M ethanolamine pH 8 (quenching solution), and further incubated for 2 h with this solution. Uncoupled antibodies were removed with 3X wash in 0.58% acetic acid in 150 mM NaCl. The beads were then transported into Eppendorf tubes.

### Immunoprecipitation

SCC47 cells plated in 150 mm dishes were serum-starved overnight, then treated with Sym004 (3 µg/ml or 30 µg/ml) for 5–120 min, or with EGF (50 ng/ml) for 5 min. The cells were then washed 2X with ice-cold PBS and lysed with mass spectrometric lysis buffer (30 mM Trizma base, 120 mM NaCl, 2 mM EDTA, 2 mM KCl, 10% glycerol, 1% DDM with phosphatase and protease inhibitors) and centrifuged (30 min, 17,000x g, 2 °C). The supernatant was then subjected for immponoprecipitation. 3 mg of cell lysate was incubated overnight at 4 °C with 60 µl of a 50% slurry of cross-linked beads. The next day, the beads were washed 5X with wash buffer (30 mM Trizma base, 120 mM NaCl, 2 mM EDTA, 2 mM KCl, 10% glycerol, 0.2% DDM with phosphatase and protease inhibitors), and the proteins were eluted with 55 µl of 1X LDS Sample buffer with Reducing agent. 5 µl was then subjected to immunoblotting, and the remaining 50 µl was separated on a gel and subjected to further processing.

### In-gel digestion

For in-gel digestion^61^, the samples were processed as described previously^62^: the IP eluates were loaded into a pre-cast SDS-PAGE gel (4–15% Mini-PROTEAN TGX Precast Protein Gel, 10-well, 50 µL) and proteins were only run for approximately 2 cm to minimise protein separation. Protein bands were excised and diced, and proteins were reduced with 5 mM TCEP in 50 mM triethylammonium bicarbonate (TEAB) at 37 °C for 20 min, alkylated with 10 mM chloroacetamide in 50 mM TEAB at ambient temperature for 20 min in the dark. Proteins were then digested with 100–150 ng trypsin, depending on the density of the band, at 37 °C for 4 h then overnight at room temperature. After digestion, peptides were extracted with acetonitrile and 50 mM TEAB washes. Samples were evaporated to dryness at 30 °C and resolubilised in 0.1% formic acid.

### LC-MS/MS

nLC-MS/MS was performed on a Q Exactive Orbitrap Plus interfaced to a NANOSPRAY FLEX ion source and coupled to an Easy-nLC 1200 (Thermo Scientific), as described previously^62^. Specifically, thirty percent of each sample was analysed as 6 µL injections with each sample analysed in triplicate technical replicates. Peptides were separated on a 24 cm fused silica emitter, 75 μm diameter, packed in-house with Reprosil-Pur 200 C18-AQ, 2.4 μm resin (Dr. Maisch) over 90 min using a linear gradient (5 to 40% B) (buffer A: 0.1% formic acid in water; buffer B: 80% acetonitrile/0.1% formic acid), at a flow rate of 250 nL/min. Peptides were ionised by electrospray ionisation using 1.8 kV applied immediately prior to the analytical column via a microtee built into the nanospray source with the ion transfer tube heated to 320 °C and the S-lens set to 50%. Precursor ions were measured in a data-dependent mode in the orbitrap analyser at a resolution of 70,000 and a target value of 3e6 ions. The ten most intense ions from each MS1 scan were isolated, fragmented in the HCD cell, and measured in the orbitrap at a resolution of 17,500.

### Protein and phosphosite data analysis

Raw data were analysed as described previously^62^ with MaxQuant^63^ version 1.5.2.8 where they were searched against the human UniProt database (http://www.uniprot.org/, downloaded 04/12/2018 using default settings. Carbamidomethylation of cysteines was set as fixed modification, and oxidation of methionines, acetylation at protein N-termini, phosphorylation (STY) and lysine ubiquitination (GlyGly tag) were set as variable modifications. Enzyme specificity was set to trypsin with maximally 2 missed cleavages allowed. To ensure high confidence identifications, PSMs, peptides, and proteins were filtered at a less than 1% false discovery rate (FDR). Label-free quantification in MaxQuant was used with a match time window of 0.7 min, an alignment time window of 20 min to quantify the AP-MS data with normalisation skipped and the ‘match between runs’ feature selected.

For statistical protein quantification analysis, the ‘proteinGroups.txt’ and ‘evidence.txt’ output files from MaxQuant were loaded into the MSstats quantification package^64^ (version 3.14.0) run through RStudio (version 1.1.456, R version 3.5.1). Contaminants and reverse sequences were removed and data was log2 transformed. To find differentially abundant proteins across conditions, significance analysis consisting of fitting a statistical model and performing a model-based comparison of conditions was carried out. The group comparison function was employed to test for differential abundance between conditions. p values were adjusted to control for the false discovery rate using the Benjamini-Hochberg procedure^65^. Quantified proteins were annotated with data from the Contaminant Repository for Affinity Purification Mass Spectrometry Data (CRAPome)^66^ to use as a filter.

As EGFR was detected in the IgG sample, proteins found in the IgG sample could not be instantly removed. Proteins present with a 100-fold or more fold change in the EGF treated sample as compared to the IgG sample (adj.p-value ≤ 0.05), as well as proteins solely detected in the EGF or IgG sample or not detected in neither sample were kept. Proteins found in less than 20% of experiments in the CRAPome database were kept. All other proteins were filtered out. Protein regulation between conditions was defined with the following cutoffs: ± 2 fold change, adj.p-value ≤ 0.05, or if they were completely absent from one of the conditions tested. Protein quantification was summarised with the MSstats function “quantification”, with type set to “Group”. The heatmap of these quantities was plotted using the pheatmap R package. The rows were clustered with the default hierarchical clustering according to Euclidean distance. NA values are shown in light grey.

### Sub-cellular fractionation

The assay was performed as described previously^67^ with modifications. Briefly, the cells (plated at 6x10^6^ cells/150 mm dish 24 h prior in complete medium) were scraped on ice into hypotonic lysis buffer (HEPES-KOH 10 mM, pH 7.2, sucrose 0.25 M, EDTA 1 mM, MgOAc 1 mM with protease and phosphatase inhibitors), and the membranes were fragmented with subsequent passing the cells 5–10 times through a 21 N and a 25 N needle. The lysates were centrifuged twice for 10 min at 1000xg to remove nuclei. Plasma membrane fractions were pelleted following 10 min centrifugation at 10,000xg, and washed twice with hypotonic buffer. The remaining supernatant was centrifuged twice for 1 h at 100,000xg to separate cytosolic fraction (supernatant) from the pelleted endosomal fraction.

### Cell growth inhibition assay

For cell growth inhibition assay^68^, the cells in complete medium (*i.e*. non-starved) were treated with indicated concentrations of Sym004 for 72 h or erlotinib for 96 h, then fixed with ice-cold 10% trichloroacetic acid (Sigma-Aldrich). Following washes with dH_2_O and drying, the cells were stained with 0.4% sulforhodamine B (Sigma-Aldrich) in 1% acetic acid (Fisher Scientific). Following washes with 1% acetic acid and drying, the dye was dissolved with 10 mM Trizma base pH 10.5 (Sigma-Aldrich). The optical density (OD) was measured at 540 nm using Varioskan LUX Multimode Plate Reader (Thermo Fisher Scientific). Growth inhibition was calculated as percentage of control cell growth (corrected for OD at day 0) subtracted from 100.

### Colony formation assay

The cells in complete medium were treated with cetuximab or Sym004, both at 2.5 µg/ml or 10 µg/ml. After 24 h, the medium was replaced for fresh medium with/without cisplatin (1 µM or 2 µM). After another 24 h, the cells were trypsinised and counted, and re-plated in 100 mm dishes. The cells were allowed to form colonies for 7 days (HN5 cells) or 14 days (SCC47 and PCI30 cells). The colonies were fixed with methanol (Fisher Scientific) and acetic acid (3:1), and stained with 0.5% crystal violet (Sigma-Aldrich) in 20% methanol. Different numbers of cells were plated for each cisplatin concentration (0–2 µM) due to cisplatin toxicity. The colonies were counted manually and adjusted for the differences in plating densities.

### Statistical analysis

At least three independent experiments were performed for all immunoblotting and confocal microscopic quantifications. In confocal image analyses, at least three images were quantified per condition within each independent experiment. For TIRFM colocalisation, 23 (EGF) or 73 (Sym004) individual data points (cells) were analysed. Throughout the manuscript, two-tailed student t-test was performed when comparing two groups, and one-way ANOVA when three or more groups were compared. In clonogenic assay, one-way ANOVA was performed within each cisplatin group (untreated, 1 µM and 2 µM cisplatin). *p < 0.5; **0.01 < p < 0.05; ***p < 0.001. For mass spectrometric analysis of EGFR interactome, one experiment was performed with three technical replicates used for statistical analysis (see detailed description in ‘Protein and phosphosite analysis’ section).

## Supplementary information

Supplementary Information.

## Data Availability

The mass spectrometry proteomics data have been deposited to the ProteomeXchange Consortium vie the PRIDE^69^ partner repository with the dataset identifier PXD016208.
