# Supplementary material for: Targeting of EGFR by a combination of antibodies mediates unconventional EGFR trafficking and degradation
Source: Sci Rep. 2020 Jan 20;10:663. doi: 10.1038/s41598-019-57153-9 (PMC6970994; doi:10.1038/s41598-019-57153-9)
Supplement: Supplementary file 1 — Supplementary Information. [file 41598_2019_57153_MOESM1_ESM.pdf]

**Supplementary Information**

# Targeting of EGFR by a combination of antibodies mediates unconventional EGFR trafficking and degradation

Sylwia Jones[1], Peter J. King[1], Costin N. Antonescu[2], Michael G. Sugiyama[2], Amandeep Bhamra[3], Silvia Surinova[3], Nicos Angelopoulos[3], Michael Kragh[4], Mikkel W. Pedersen[4], John A. Hartley[1], Clare E. Futter[5] and Daniel Hochhauser*[1]

[1]Cancer Research UK Drug-DNA Interactions Research Group, UCL Cancer Institute, Paul O'Gorman Building, University College London, London WC1E 6DD, UK

[2]Department of Cell Biology, Ryerson University, Toronto, Canada

[3]Proteomics Research Core Facility, UCL Cancer Institute, University College London, London, UK.

[4]Symphogen A/S, Ballerup, Denmark

[5]UCL Institute of Ophthalmology, University College London, 11-43 Bath Street, London EC1V 9EL, UK

*Correspondence to Daniel Hochhauser: d.hochhauser@ucl.ac.uk

**Supplementary Figure S1. Sym004-Gold subcellular distribution *via* electron microscopy. Non-specific binding of cetuximab and Sym004 to secondary antibodies, but not directly-labelled primary antibodies. EGFR DIF localisation in different head and neck cancer cell lines. (a)** Quantification of colocalisation between Sym004 or EGF and clathrin was performed as described in Methods. ***p<0.001. **(b)** SCC47 cells were serum-starved and treated with Sym004-10 nm gold for 2 h or 4 h before processing for electron microscopy. After 2 h, gold was found in patches (arrows) on the limiting membrane of MVBs and also on the ILVs. After 4 h, gold was also found within electron dense lysosomes (LYS). **(c)** SCC47, HN5 and PCI30 cells were serum-starved and treated with Sym004 or EGF for 2 h, then lysed with CellLytic M. DIF, detergent-insoluble fraction. All immunoblots were cropped for clarity.

**Supplementary Figure S2. EGF and Sym004 colocalise within the same LEs/MVBs. Sym004 promotes transient activation of MAPK/Erk signalling. Cbl ligases are dispensable for Sym004-mediated EGFR degradation. (a)** SCC47 cells were serum-starved, the final 1 h in the presence of Bafilomycin A1 (600 nM). The medium was replaced for cell-imaging medium (CIM), and live-cell imaging was performed upon addition of EGF-AF647 and Sym004-AF488 (added 5 min after EGF). The images were acquired every 2 min with 24 z-stacks with 0.4 µm intervals, starting 10 min after Sym004 treatment (thus 15 min after EGF stimulation). **(b)** SCC47 were serum-starved and treated with Sym004 or EGF for indicated times, then lysed. EGFR was immunoprecipitated with anti-EGFR antibody, while normal mouse IgG was used in Control. IgG heavy chain (HC) and light chain (LC) are shown. **(c)** A431NS were serum-starved and treated with Sym004 (10 µg/ml), cetuximab (10 µg/ml) or EGF (1 nM) for indicated times, then lysed. **(d)** SCC47 cells, either serum-starved or in complete medium, were treated with cetuximab or Sym004 for indicated times, then lysed. **(e)** cCbl- or Cbl-b-depleted (or control) SCC47 cells were serum-starved and treated with Sym004 (3 µg/ml or 30 µg/ml) or EGF for 6 h, then lysed. EGFR degradation was quantified as remaining EGFR (normalised to tubulin) relative to untreated cells. Error bars, SEM. *p<0.05. All immunoblots were cropped for clarity.

**Supplementary Figure S3. Depletion of Alix, Rab11 or Vps35 does not affect EGFR internalisation, degradation or plasma membrane localisation. (a)** Alix-depleted (or control) SCC47 cells were serum-starved and treated with Sym004 (3 µg/ml or 30 µg/ml) or EGF for 6 h, then either lysed (for immunoblotting) or fixed and stained with EGFR-AF568 (for immunofluorescence). **(b)** Vps35 or Rab11-depleted (or control) SCC47 cells were serum-starved and treated with Sym004 (3 µg/ml or 30 µg/ml) for 6 h, then lysed. **(c)** Vps35 or Rab11-depleted (or control) SCC47 cells were serum-starved and treated as in A, then fixed and stained with EGFR-AF568. Hoechst 33342 was used to stain the nuclei. All immunoblots were cropped for clarity.

**Supplementary Figure S4. Additional phosphorylated residues identified within EGFR.** Presented are all phosphorylated residues identified within EGFR in addition to the residues shown in Figure 3D.

**Supplementary Figure S5. Both p38 activity and EGFR TK activity are dispensable for EGFR endocytosis/degradation in HNC cells. (a)** PCI30 and HN5 cells were serum-starved, the final 30 min in the presence of erlotinib. The cells were then treated with Sym004 or EGF for 6 h, then lysed. EGFR degradation was quantified as remaining EGFR normalised to untreated cells. Error bars, SEM. **(b)** PCI30 and HN5 cells were treated as in B, then fixed and stained with EGFR-AF488 and Phalloidin-AF647 antibodies. Hoechst 33342 was used to stain the nuclei. **(c)** Steady-state (non-starved) SCC47, PCI30 and HN5 cells were lysed. Relative EGFR level (normalised to αtubulin) was quantified as a fold difference. Error bars, SEM. **(d)** SCC47 cells were serum-starved overnight, final 30 min in the presence of p38 inhibitor SB203580 (10 µM), then treated with cetuximab, Sym004, EGF or cisplatin (50 µM or 200 µM) for 6 h. Cell-surface proteins were then biotinylated (detailed in Methods), following by cell lysis. All immunoblots were cropped for clarity.

**Supplementary Figure S6. Sym004-mediated EGFR endocytosis does not proceed through lipid raft formation (a)** AP2α- or caveolin-1-depleted (or control) SCC47 cells were serum-starved and treated with Sym004 (3 µg/ml or 30 µg/ml) or EGF for 6 h, then lysed. EGFR degradation was quantified as remaining EGFR normalised to untreated cells. Error bars, SEM. **(b)** AP2α1- or caveolin-1-depleted (or control) SCC47 cells were treated as in A, then fixed and stained with EGFR-AF488 antibody. **(c)** AP2α1- or clathrin-depleted (or control) SCC47 cells were serum-starved, then incubated with Transferrin-AF568 (50 µg/ml) for 30 min and fixed. Hoechst 33342 was used to stain the nuclei. **(d)** Flot1- or Flot2-depleted (or control) SCC47 cells were serum-starved and treated with Sym004 (3 µg/ml or 30 µg/ml) or EGF for 6 h, then lysed. All immunoblots were cropped for clarity.

**Supplementary Figure S7. K44 dynamin does not prevent Sym004 endocytosis, which also differs from macropinocytic uptake of dextran. (a)** SCC47 cells transfected with wild-type or mutant dynamin-eGFP were serum-starved and treated with Sym004-AF568 or EGF-AF647 for indicated times, then fixed. **(b)** SCC47 cells were treated with cytochalasin D as indicated, or DMSO (control) for 1 h, then fixed and stained with Phalloidin-AF633. **(c)** SCC47 cells were serum-starved and treated with Fluorescein-conjugated dextran and Sym004-AF488 or EGF-AF647 for 10 min or 30 min, then fixed. Hoechst was used to stain the nuclei. Arrowheads indicate colocalisation between Sym004 or EGF and dextran. Hoechst 33342 was used to stain the nuclei.

**Supplementary Figure S8. Sym004 mediates EGFR degradation in three HNC cell lines. HNC cells with high EGFR membrane expression exhibit lower endosomal-to-membrane EGFR ratio. (a)** SCC47, HN5 and PCI30 cells were serum-starved and treated with Sym004, EGF or both for indicated times, then

lysed. **(b)** SCC47, PCI30 and HN5 cells were treated as in Figure 6C. Sym004 membrane binding was quantified as explained in Methods. **(c)** Representative images of colonies for Figure 6F. All immunoblots were cropped for clarity.

**Supplementary Figure S9. Uncropped immunoblots related to Figures 1-6.**

Supplementary Figure S1

a

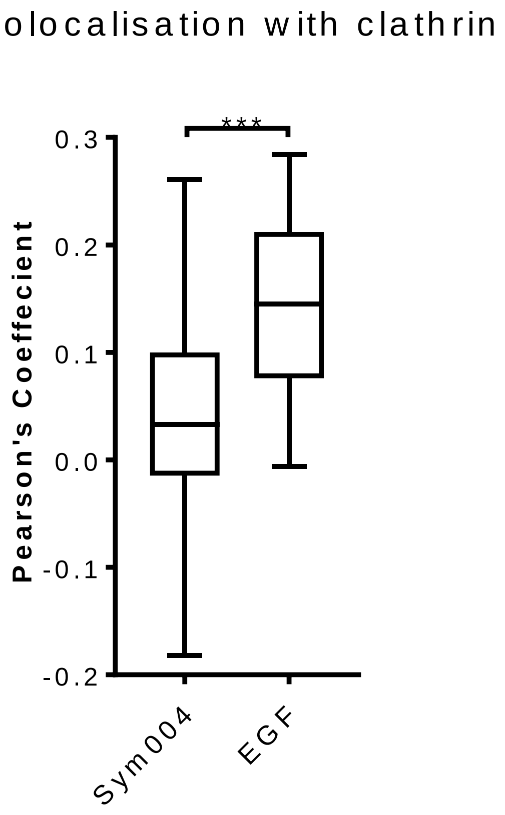


b

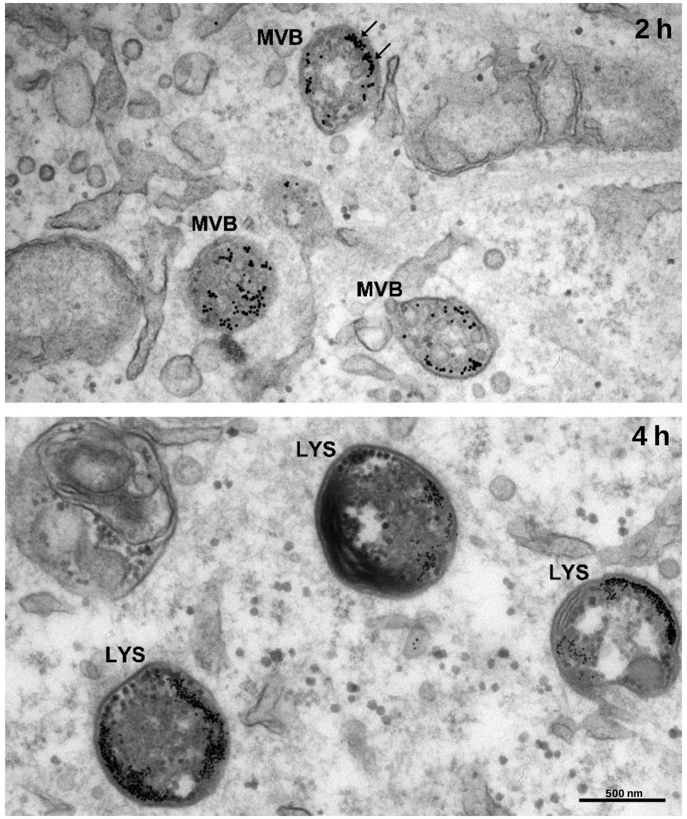


c

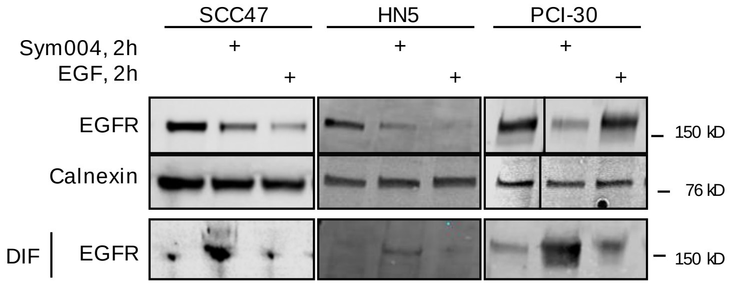

# Supplementary Figure S2

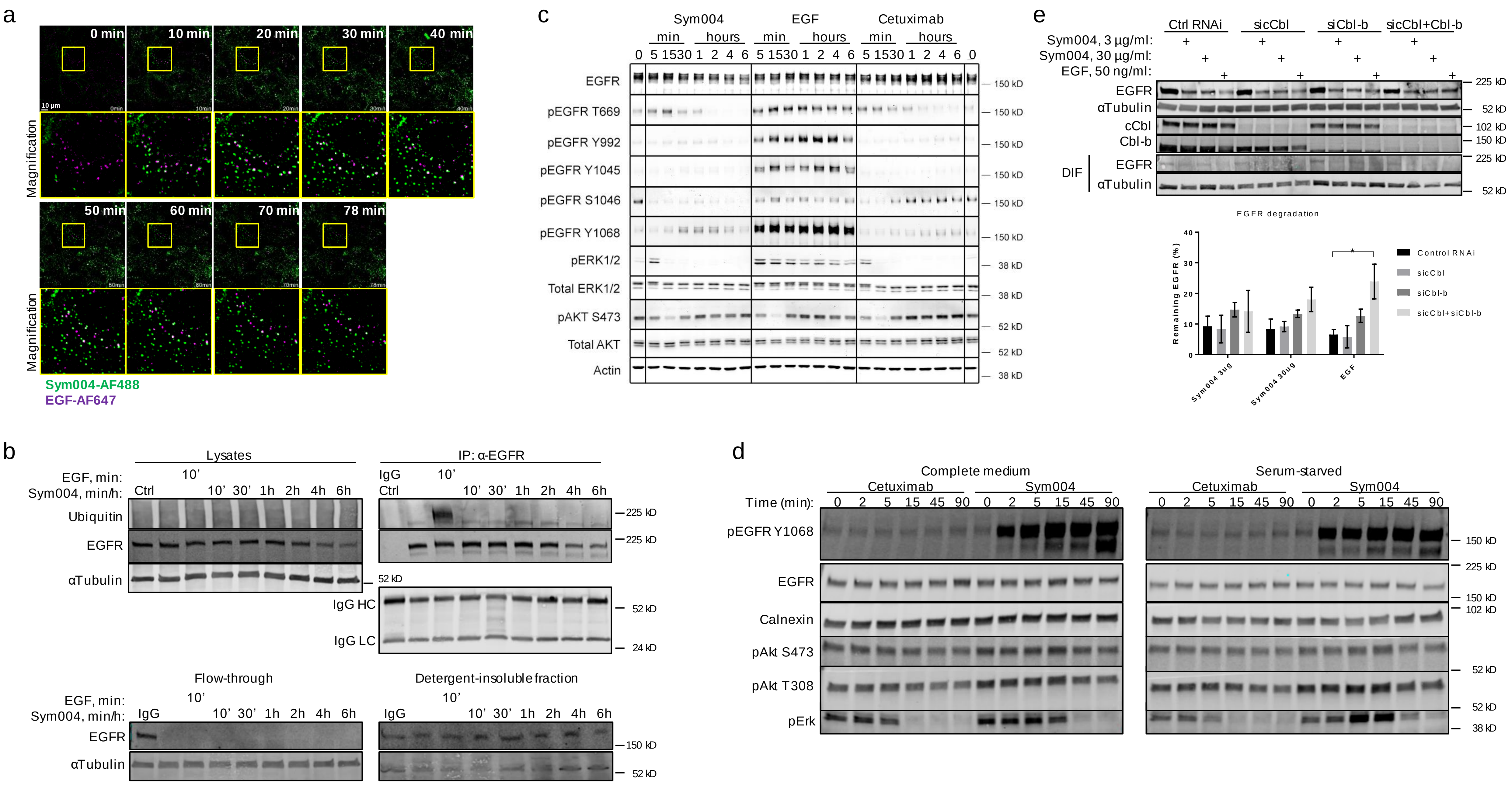

Supplementary Figure S3

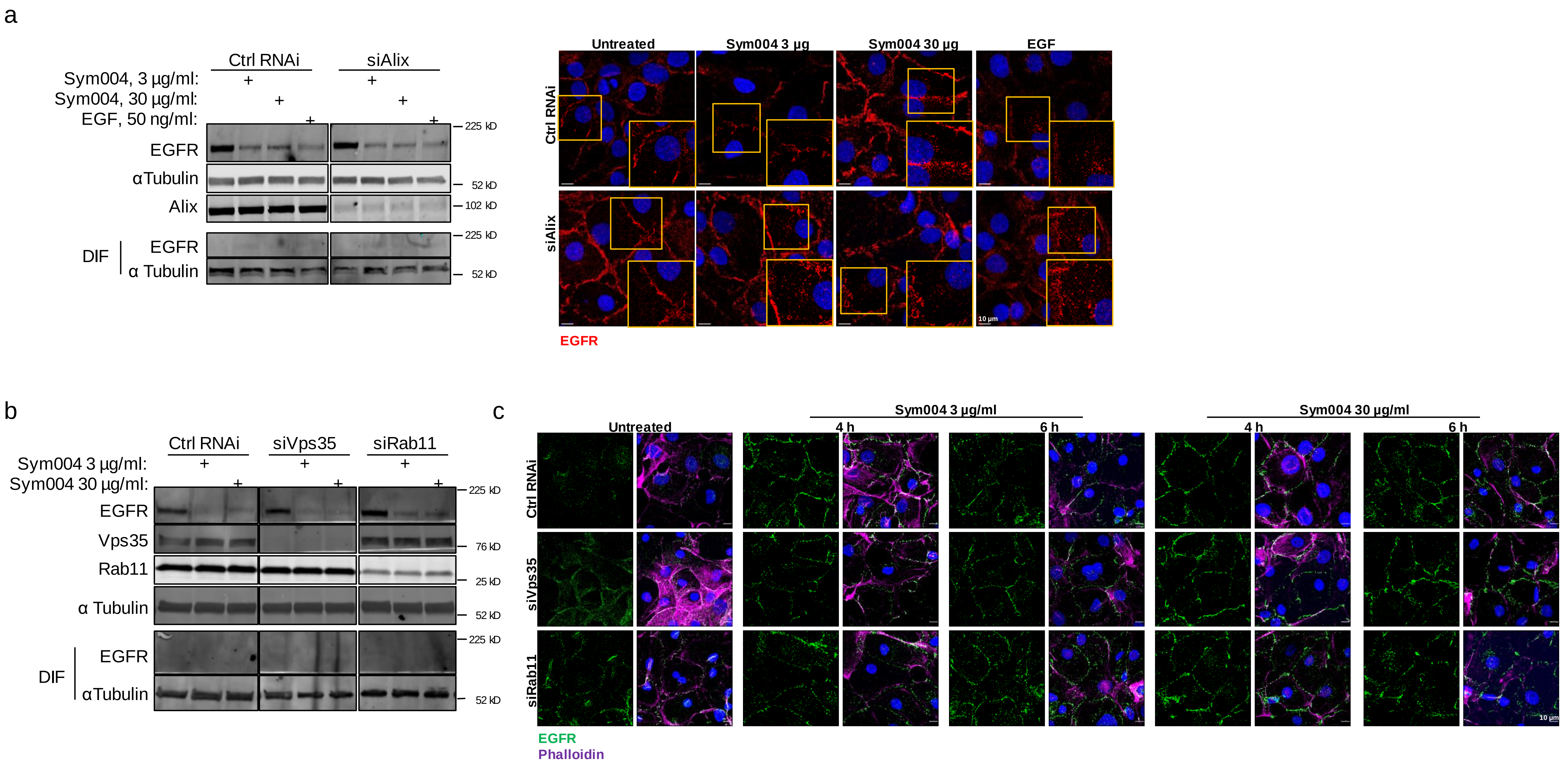

# Supplementary Figure S4

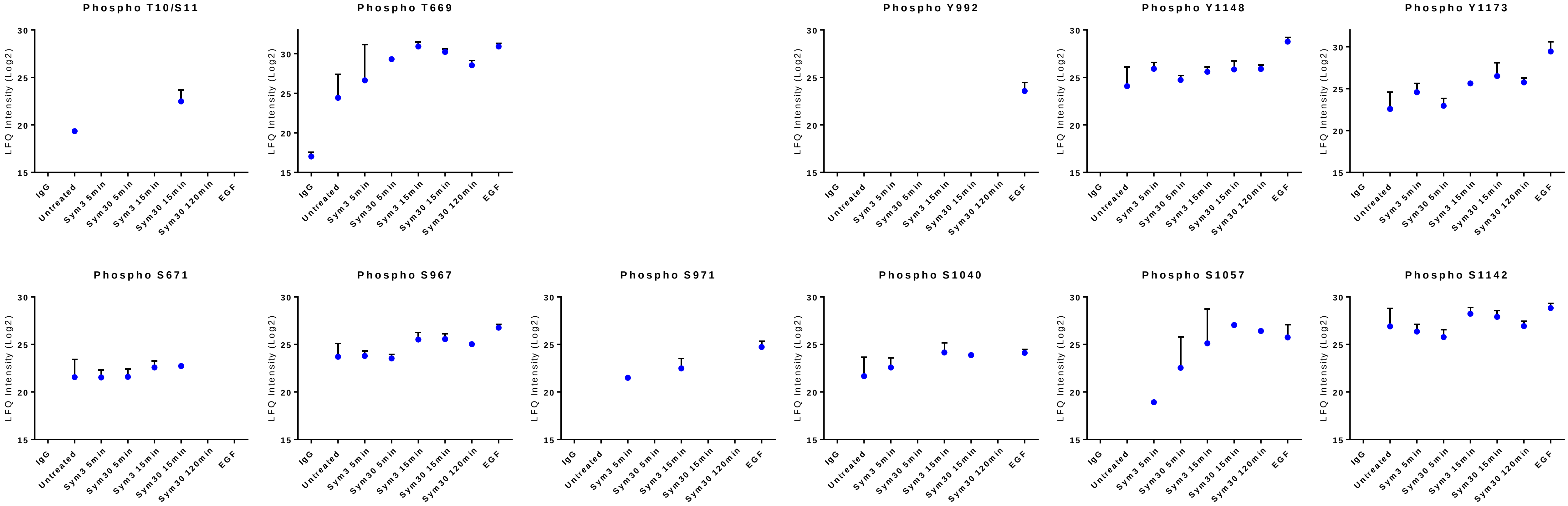

# Supplementary Figure S5

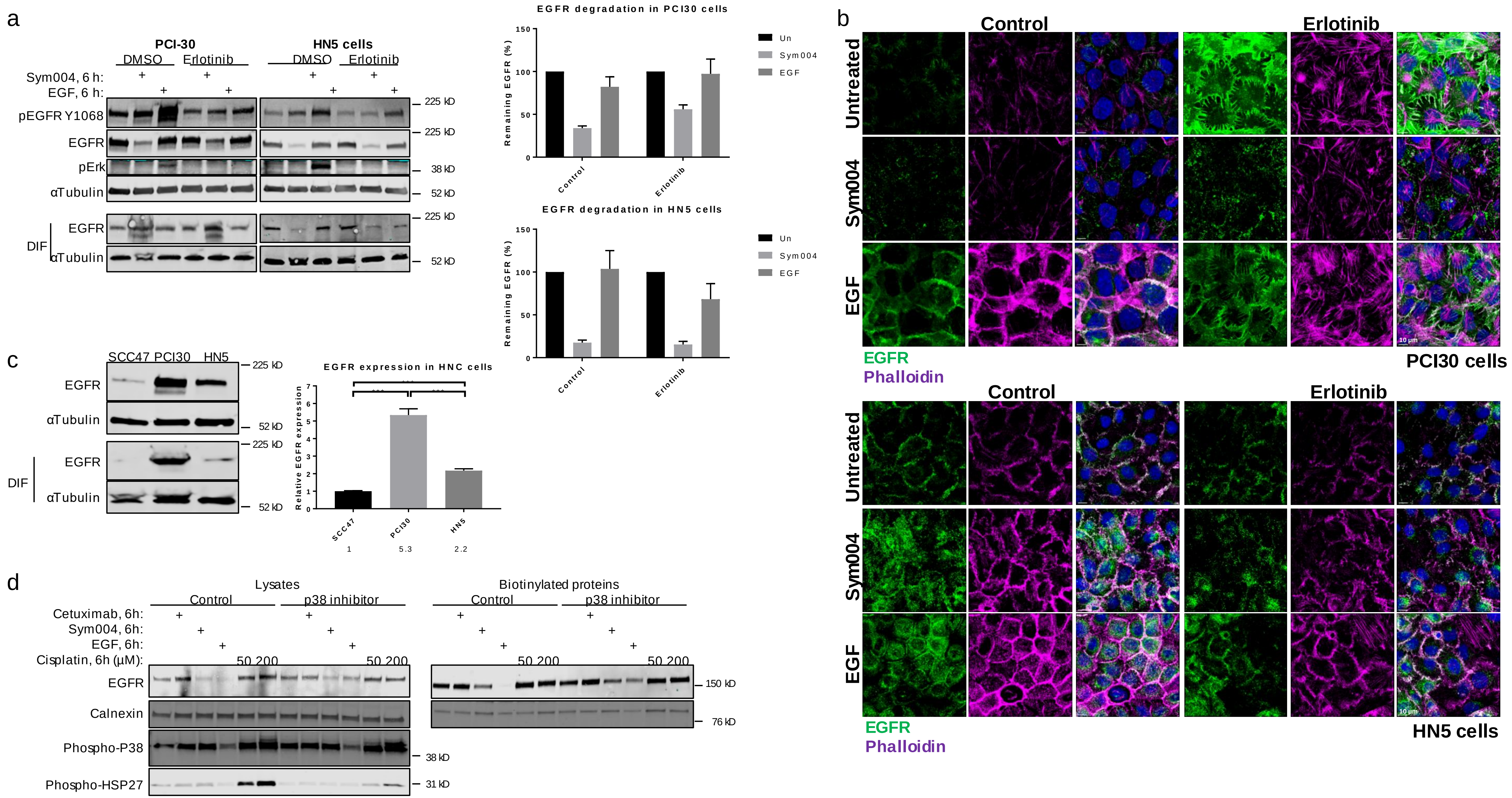

# Supplementary Figure S6

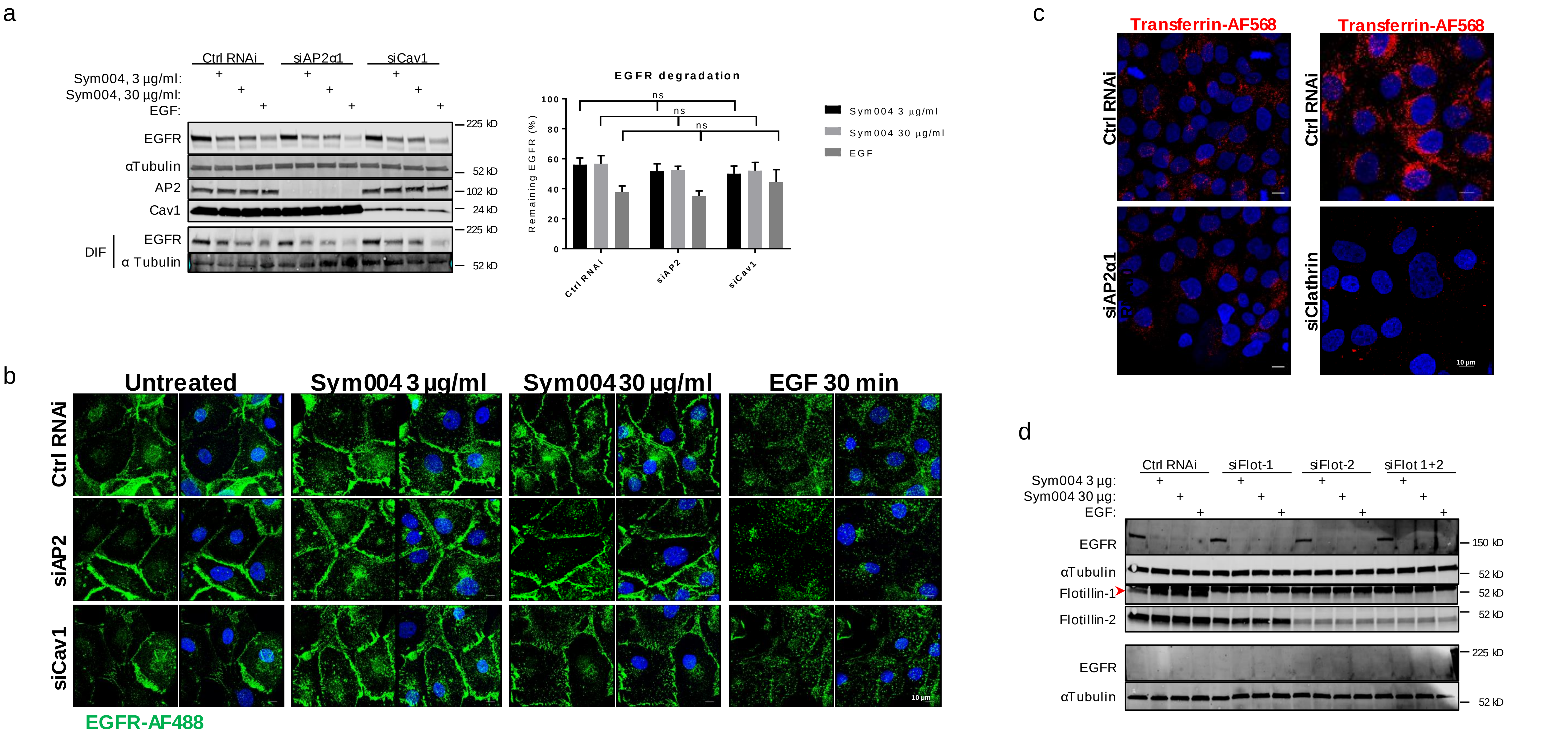

Supplementary Figure S7

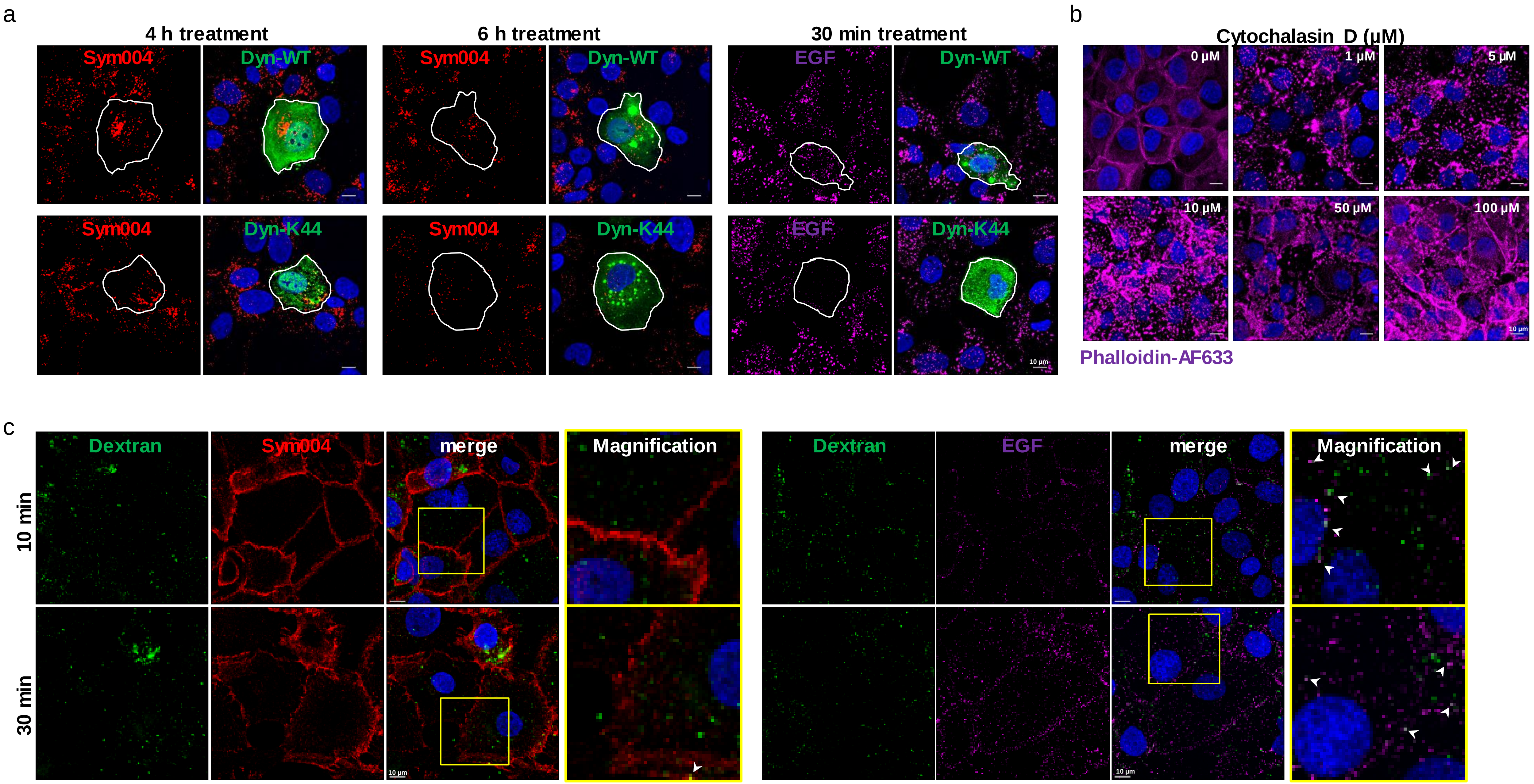

# Supplementary Figure S8

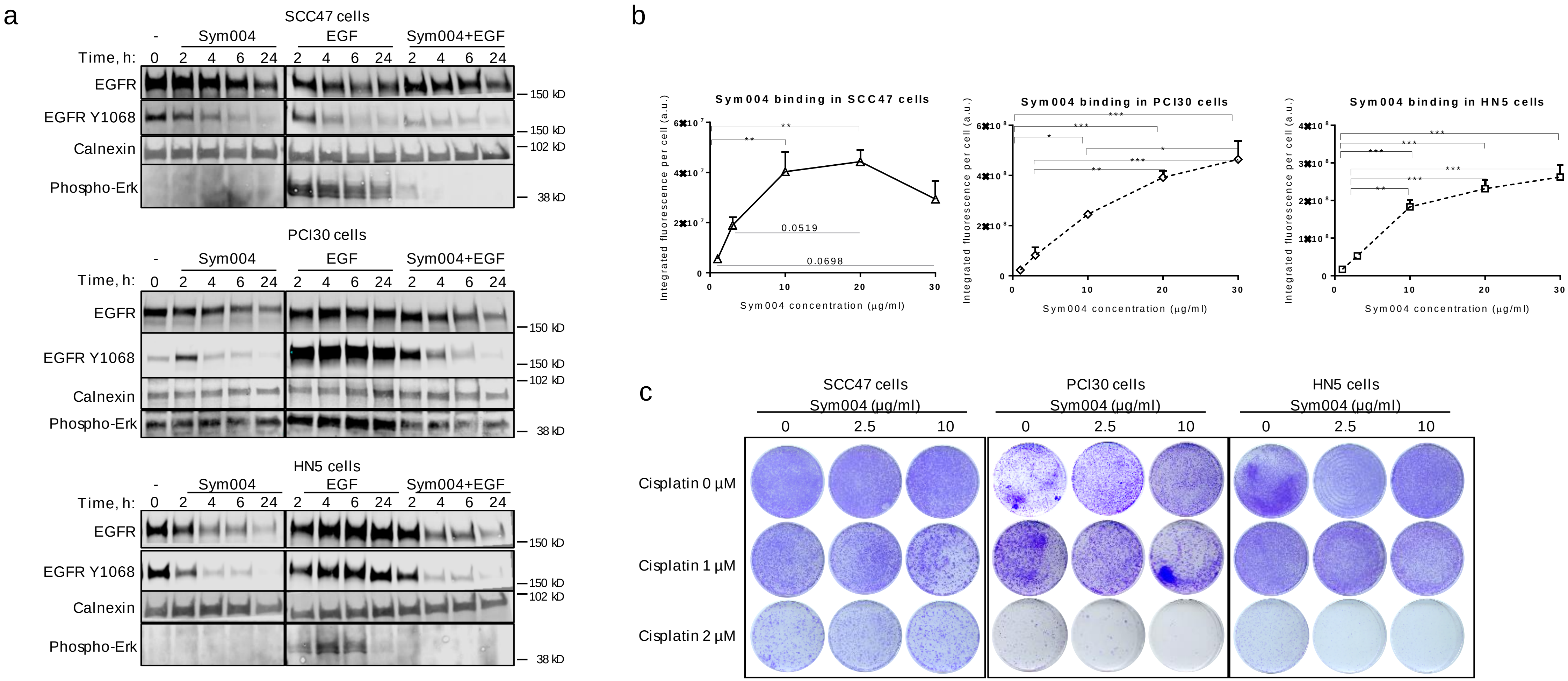

# Supplementary Figure S9

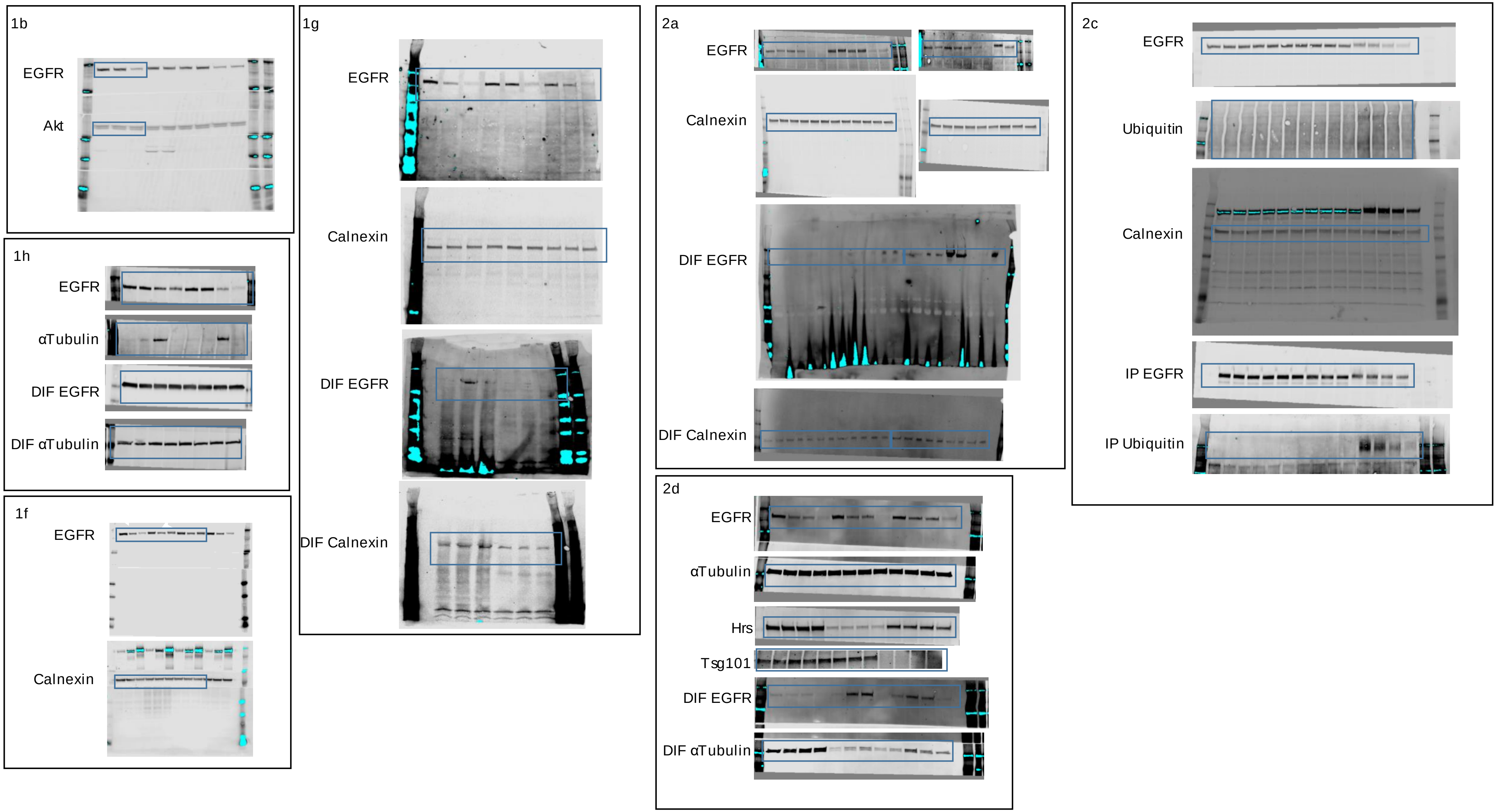

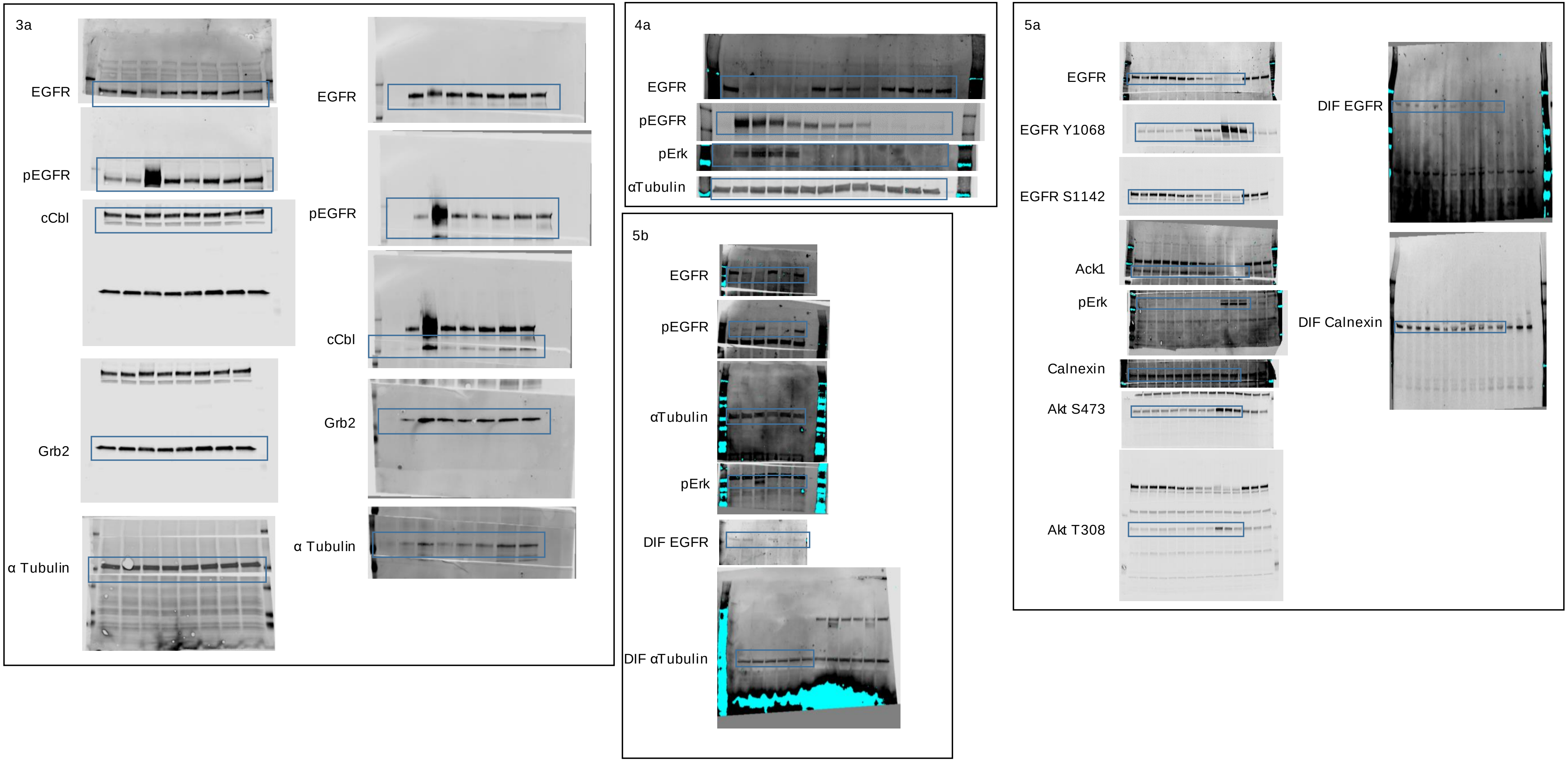
3a
EGFR
pEGFR
cCbl
Grb2
α Tubulin
EGFR
pEGFR
cCbl
Grb2
α Tubulin
4a
EGFR
pEGFR
pErk
αTubulin
5b
EGFR
pEGFR
αTubulin
pErk
DIF EGFR
DIF αTubulin
5a
EGFR
EGFR Y1068
EGFR S1142
Ack1
pErk
Calnexin
Akt S473
Akt T308
DIF EGFR
DIF Calnexin

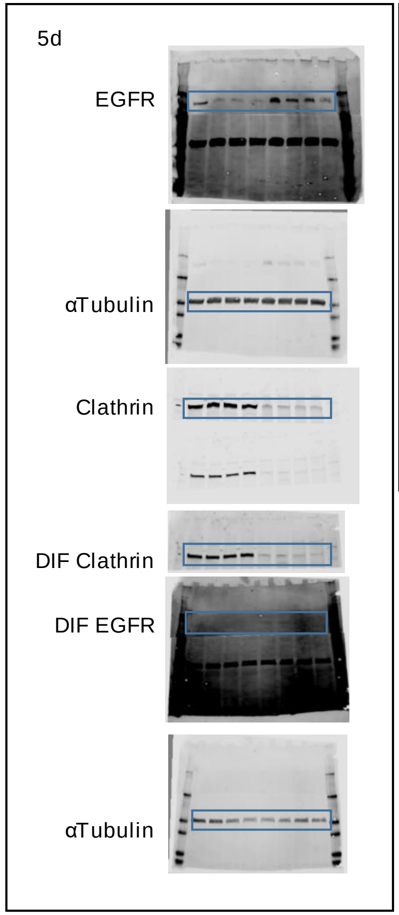
5d
EGFR
αTubulin
Clathrin
DIF Clathrin
DIF EGFR
αTubulin

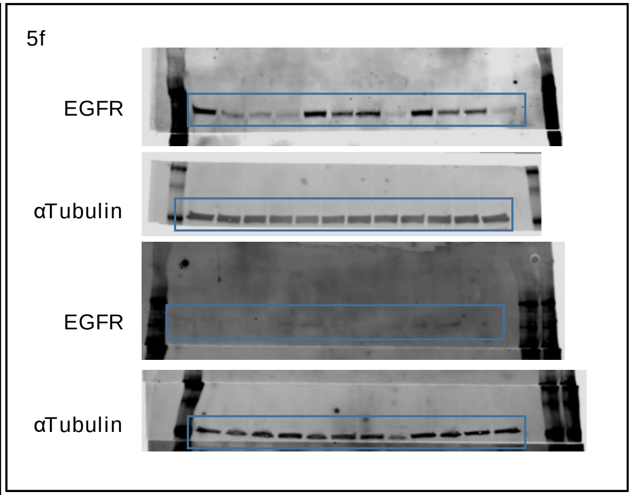
5f
EGFR
αTubulin
EGFR
αTubulin

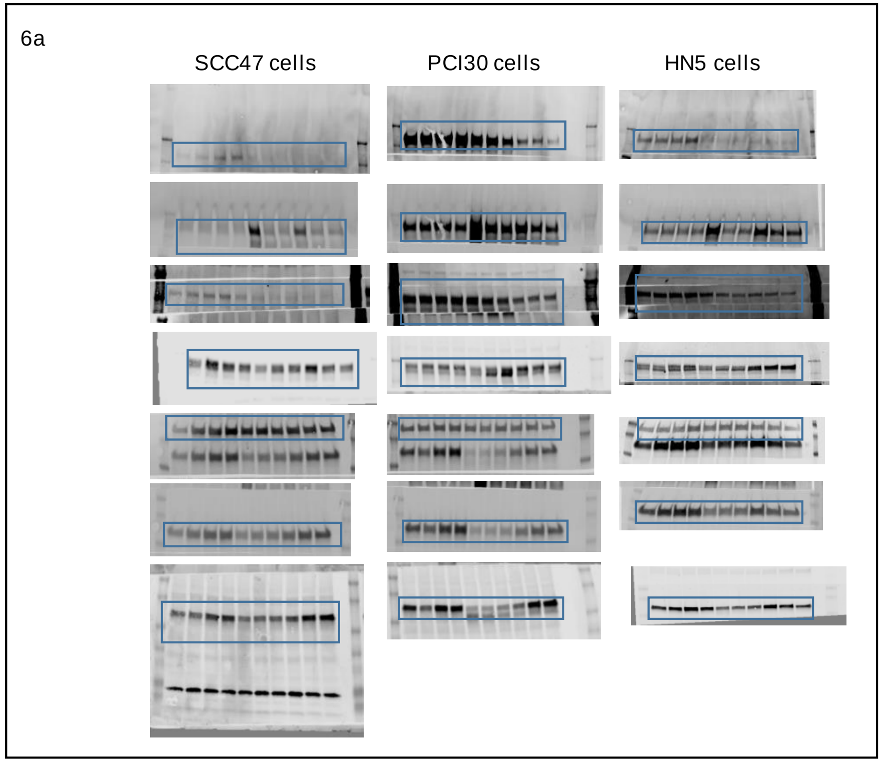
6a
SCC47 cells
PCI30 cells
HN5 cells

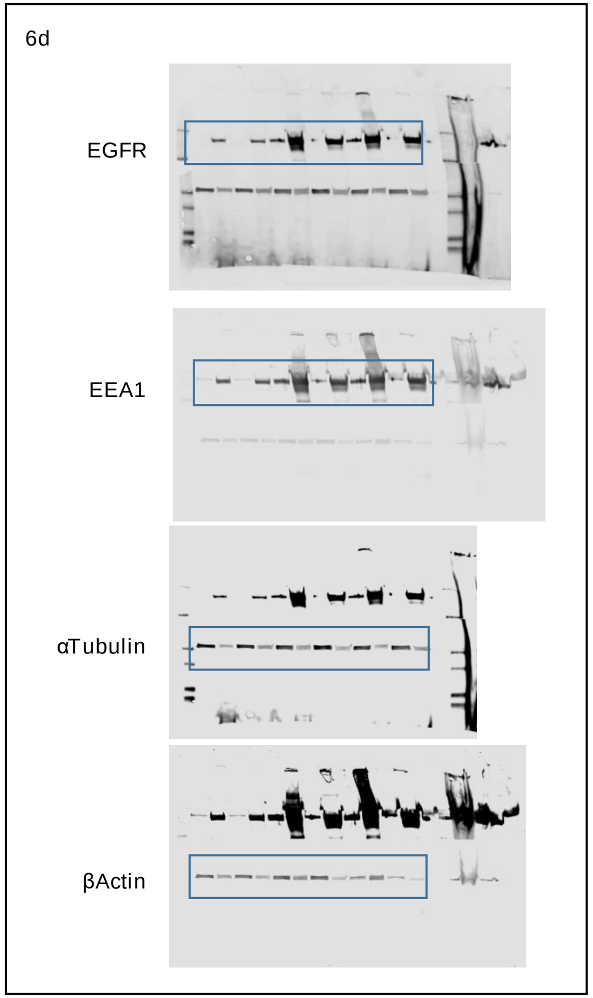
6d
EGFR
EEA1
αTubulin
βActin
